# Genetic Variability and Phylogenetic Analysis of Han Population from Guanzhong Region of China based on 21 non-CODIS STR Loci

**DOI:** 10.1038/srep08872

**Published:** 2015-03-09

**Authors:** Yu-Dang Zhang, Xiao-Li Tang, Hao-Tian Meng, Hong-Dan Wang, Rui Jin, Chun-Hua Yang, Jiang-Wei Yan, Guang Yang, Wen-Juan Liu, Chun-Mei Shen, Bo-Feng Zhu

**Affiliations:** 1 Research Center of Stomatology, Stomatological Hospital, Xi'an Jiaotong University, Xi'an, 710004, P. R. China; 2 Xi'an Jiaotong University Health Science Center, Xi'an 710061, P. R. China; 3 Department of Biochemistry, Medical college of Nanchang University, Nanchang. 330001, P. R. China; 4 Medical Genetic Institute of Henan Province, Henan Provincial People's Hospital, People's Hospital of Zhengzhou University, Zhengzhou 450003, P. R. China; 5 Department of Radiology, The Second Affiliated Hospital of Xi'an Jiaotong University, Xi'an. 710004, P. R. China; 6 People's hospital of Arong Banner, Hulun Buir City. 162750, P. R. China; 7 Key Laboratory of Genome Sciences, Beijing Institute of Genomics, Chinese Academy of Sciences, Beijing 100101, P. R. China; 8 Department of Pathology and Laboratory Medicine Loma Linda University Medical Center, California. 92354, USA; 9 College of Life Sciences, Shaanxi Normal University, Xi'an. 710062, P. R. China

## Abstract

In the present study, we presented the population genetic data and their forensic parameters of 21 non-CODIS autosomal STR loci in Chinese Guanzhong Han population. A total of 166 alleles were observed with corresponding allelic frequencies ranging from 0.0018 to 0.5564. No STR locus was observed to deviate from the Hardy-Weinberg equilibrium and linkage disequilibriums after applying Bonferroni correction. The cumulative power of discrimination and probability of exclusion of all the 21 STR loci were 0.99999999999999999993814 and 0.999998184, respectively. The results of genetic distances, phylogenetic trees and principal component analysis revealed that the Guanzhong Han population had a closer relationship with Ningxia Han, Tujia and Bai groups than other populations tested. In summary, these 21 STR loci showed a high level of genetic polymorphisms for the Guanzhong Han population and could be used for forensic applications and the studies of population genetics.

China is an ancient country with 5,000-year-long civilization and has the largest population in the world, about 1.371 billion in the sixth national population census of China in 2010. As the biggest one of the 56 ethnic groups and with a population of approximately 1.226 billion, the Han population is widespread across China. Their spoken and written language is Chinese, one branch of the Sino-Tibetan language family. Chu et al. constructed the phylogenies using the neighbor-joining method based on difference population data for short tandem repeat (STR) loci and concluded that there was the distinction between southern and northern populations in China^1^. For Chinese Han population, previous population genetic studies based on STRs or single nucleotide polymorphisms (SNPs) have shown that the Chinese Han population was intricately sub-structured and clustered roughly to two (northern Han and southern Han)^2^,^3^ or three (northern Han, central Han and southern Han) subgroups^4^. So, it is of significance to further clarify the genetic structure of Chinese Han populations from different regions.

Guanzhong region, literally means “within the passes” in Chinese, is located in the middle of the Chinese mainland and includes the cities of Xi'an, Tongchuan, Baoji, Xianyang and Weinan in Shaanxi province, China. There are several ethnic groups, mainly including Han, Hui and Manchu nationalities living together in the region. Shen et al. reported that the Guanzhong Han population had the close genetic relationship with the northern and southern Han populations using genetic distance measurements, neighbor-joining dendrograms and principal component analysis (PCA) base on different HLA loci^5^.

STRs have been the most widely used in forensic science and population genetics. In order to provide more genetic information and increase the power of discrimination (PD) and probability of exclusion (PE), more novel STR loci with high genetic polymorphisms were integrated into one fluorescence-labeled multiplex amplification system. And, it is necessary to analyze the allelic distribution of STR loci before used in forensic applications. We have so far reported population data^6^,^7^,^8^,^9^,^10^,^11^,^12^,^13^,^14^ for a panel of 21 STR loci, and these STR loci demonstrated tremendous potential for forensic applications. In the present study, we first aimed to present the population genetic data and forensic parameters of the Chinese Guanzhong Han (Northern Han in geography) with a panel of 21 non-CODIS autosomal STRs. Moreover, we investigated the genetic relationships and population differentiations between Guanzhong Han and other Chinese groups.

## Methods

### Populations and DNA extraction

Blood samples were randomly collected from 275 unrelated individual of the Han Chinese living in Guanzhong region, Shaanxi province, China. Before getting involved in the study, all the participants signed the written informed consents for the sample collections and succedent analyses. This study was conducted according to the humane and ethical research principles and approved by the ethical committee of Xi'an Jiaotong University Health Science Center, China. The genomic DNA was extracted from blood-stained samples using the Chelex-100 method as described by Walsh et al.^15^.

**Figure 1:**
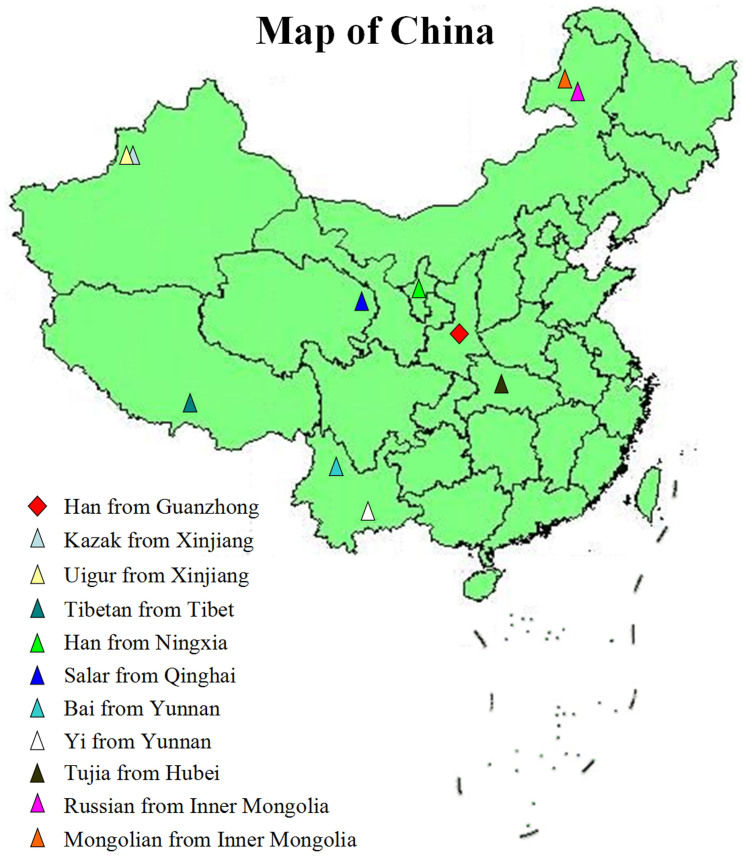
The geographical locations of the Guanzhong Han and 10 reference groups in China. The map was created in matlab R2013b software (MathWorks Inc., USA).

Genotyping results of the 21 STR loci from 10 Chinese groups were chosen for population comparison, including Mongolian (n = 86) from Inner Mongolia autonomous region^6^, Bai (n = 106) from Yunnan province^7^, Kazak (n = 114) from Xinjiang autonomous region^8^, Ningxia Han (Northern Han) (n = 202) from Ningxia autonomous region^9^, Russian (n = 114) from Inner Mongolia autonomous region^10^, Tibetan (n = 104) from Tibet autonomous region^11^, Tujia (n = 107) from Hubei province^12^, Uigur (n = 218) from Xinjiang autonomous region^13^, Yi (n = 110) from Yunnan province^14^, Salar (n = 120) from Qinghai province^16^. The geographical locations of the reference populations were shown in Figure 1.

### PCR amplification and STR typing

A panel of STRs were amplified in a single reaction using the AGCU 21+1 STR system (AGCU ScienTech Incorporation, Wuxi, Jiangsu, China), according to the manufacturer's instructions. The PCR products were separated and detected by capillary electrophoresis on the ABI 3130xl Genetic Analyzer (Applied Biosystems, Foster City, CA, USA). The STR typing results were obtained by comparing to the 21+1 Allelic Ladder using the program GeneMapper® ID-X v1.3 (Applied Biosystems, Foster City, CA, USA). Control DNA from 9947A cell line (Promega Corporation, Madison, WI, USA) was typed for quality control. All laboratory procedures were in accordance with the laboratory internal control standards.

### Statistical analyses

Allelic frequencies and forensic parameters were calculated using the modified Powerstats v1.2^17^. The Genepop v4.0.10 (http://genepop.curtin.edu.au/) was utilized to estimate the linkage disequilibriums (LDs) for all pair-wise STR loci. To estimate the inter-population differentiations between the Guanzhong Han and 10 reference populations in China, the locus-by-locus *Fst*, associated *p* and overall *Fst* values were calculated using the method of analysis of molecular variance (AMOVA) by the software ARLEQUIN v3.1 (http://cmpg.unibe.ch/software/arlequin3) and the *D_A_* distances were calculated using the DISPAN program^18^. To visually estimate the genetic relationships between the Guanzhong Han and reference populations, we performed two kinds of phylogenetic trees using the software MEGA v5 with the unweighted pair-group method with arithmetic means (UPGMA) based on *D_A_* distances and the software PHYLIP v3.6 by a bootstrap-over-loci method with 1,000 replicates based on allelic frequencies, respectively. A PCA plot was conducted with MATLAB 2007a (MathWorks Inc., USA) based on allelic frequencies of 21 STRs. The existence of significant LD among STRs has an impact on some subsequent analyses, including *D_A_* calculation and MEGA, so the STR loci which observed to be in significant LD with one or more other loci would be removed in the analyses mentioned above.

## Results and Discussion

The typing results of the 21 STR loci from the Guanzhong Han population were listed in supplemental Table 1, and the allelic frequencies and forensic parameters were shown in Table 1. A total of 166 alleles were observed with corresponding allelic frequencies in the range of 0.0018 to 0.5564. No STR locus was observed to deviate from the Hardy-Weinberg equilibrium (after Bonferroni's correction; *p* > 0.00238). All the 21 loci showed a high level of PD values, ranging from 0.7700 for D1S1627 locus to 0.9437 for D19S433 locus. The values ranged from 0.5325 (D1S1627 locus) to 0.7916 (D19S433 locus) for polymorphism information content and 0.2738 (D1GATA113 locus) to 0.5856 (D19S433 locus) for PE, respectively. Observed heterozygosity ranged from 0.5855 (D1GATA113 locus) to 0.7927 (D19S433 locus), while the expected heterozygosity ranged from 0.5940 (D1S1627 locus) to 0.8147 (D19S433 locus). The cumulative PD and PE of all the 21 STR loci were 0.99999999999999999993814 and 0.999998184, respectively. The results indicated that the panel of 21 STRs showed a high level of polymorphisms and were suited for personal identification and parentage testing in forensic science.

LD is the correlations among neighboring alleles descended from single, ancestral chromosomes^19^. The level of LD is affected by multiple factors, for example, genetic linkage, population structure and natural selection. In the present study, 11 out of 210 pairwise loci were observed to be in linkage disequilibriums for 21 STR loci in Guanzhong Han population (shown in supplementary Table 2). However, no significant linkage disequilibrium remained after applying Bonferroni correction (*p* < 0.05/210 = 0.00024). In addition, the loci previously reported to be in significant LD with other loci in the reference groups were removed in some subsequent analyses and there were 10 loci (including D10S1248, D11S4463, D14S1434, D18S853, D1GATA113, D22S1045, D2S441, D4S2408, D6S1017 and D9S1122) reserved for the analyses of *D_A_* calculation and MEGA.

Population differentiations between the Guanzhong Han and other 10 previously published groups were performed by the method of AMOVA based on the allelic frequencies of 21 STR loci. As shown in Table 2, the Guanzhong Han population was observed to be significantly different from the Uigur group at 9 loci, then from the Yi, Tibetan, Kazak, Russian and Salar groups at 3, 3, 2, 1, and 1 STR loci, respectively (after Bonferroni's correction; *p* < 0.00238). No significant difference was observed between the Guanzhong Han population and the Ningxia Han, Bai, Tujia and Mongolian groups. Nine loci, including D11S4463, D14S1434, D18S853, D19S433, D20S482, D2S1776, D4S2408, D6S1017 and D9S1122, showed no significant difference between the Guanzhong Han and reference groups. There were up to 4 reference groups at D22S1045 locus; 2 groups at D12ATA63, D1GATA113, D3S4529 and D5S2500 loci, showing significant difference from the Guanzhong Han population, respectively; and the results indicated that these loci had higher population differentiation and were appropriate for the studies of inter-population comparison.

The *D_A_* distance values based on the 10 loci between the Guanzhong Han and 10 reference groups were shown in Table 3. The largest *D_A_* distance (0.0337) was observed between the Guanzhong Han and the Yi group, followed by Russian (0.0281) and Salar (0.0264) groups; whereas the smallest distance was found with the Ningxia Han population (0.0073), followed by Tujia (0.0077) and Bai (0.0091) groups. The *D_A_* distances between Guanzhong Han and Kazak, Tibetan, Mongolian and Uigur groups were 0.0126, 0.0133, 0.0141 and 0.0153, respectively. The *D_A_* distances showed closer relationship between the Guanzhong Han and the Ningxia Han, Tujia and Bai populations. In addition, the population differentiations between the Guanzhong Han and reference groups obtained from the overall *Fst* values based on all the 21 loci by the AMOVA method were basically in line with that from the *D_A_* distances.

**Figure 2:**
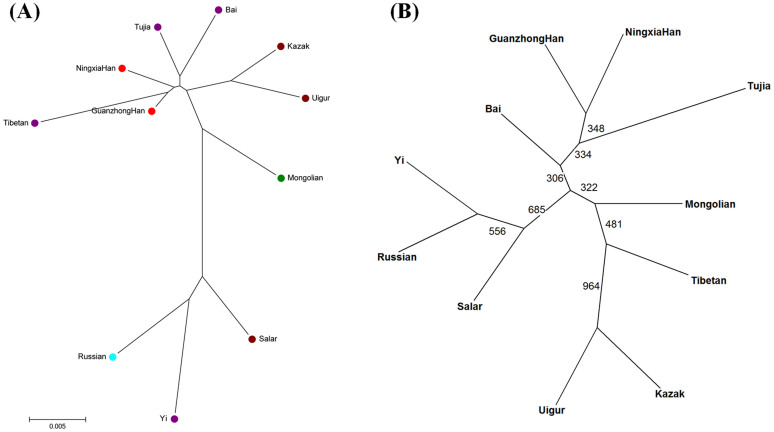
Phylogenetic tree for Guanzhong Han and 10 reference populations constructed by the software MEGA v5 based on *D_A_* distances (A) and by the software PHYLIP v3.6 based on allelic frequencies (B), respectively.

The phylogenetic tree constructed by the software MEGA v5 based on *D_A_* distances was shown in Figure 2A. From the figure, three clusters were observed: the Guanzhong Han, Ningxia Han, Tibetan, Tujia and Bai groups shared the same clade; Yi, Russian, Salar and Mongolian groups were delineated in a branch; the remaining groups including Uigur and Kazak groups clustered together. In order to further confirm the phylogenetic relationship, the phylogenetic tree was also constructed using PHYLIP v3.6 based on the allelic frequencies of 21 STR loci and the result was shown in Figure 2B. The results obtained from two phylogenetic trees were extremely similar, and the only exception was Tibetan group. The exception may due to the different number of STR loci.

**Figure 3:**
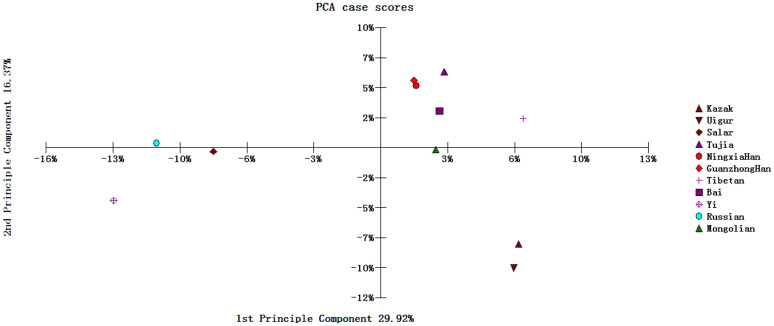
Principal component analysis plot structured based on allelic frequencies of 21 STR loci in 11 populations.

As shown in Figure 3, the PCA plot among 11 groups was obtained with the first two components to be 29.92% and 16.37%, respectively, which could explain 46.29% of the variance. The Guanzhong Han population was observed to cluster closest with the Ningxia Han population, then with the Tujia and Bai groups, which is consistent with the results of phylogenetic trees above. The genetic evidence in our study showed that the Guanzhong Han population had closer relationship with Ningxia Han, Tujia and Bai populations than other 7 groups. The present result was basically consistent with the previous result of HLA loci as described by Shen et al.^5^. In order to further understand their genetic relationships and ancestry information, more genetic markers, such as SNPs and insertion/deletion polymorphisms should be used and analyzed in future.

## Conclusions

In conclusion, we presented the genetic data of the Guanzhong Han population with 21 STR loci, and these STR loci showed high level of genetic polymorphisms and were suited for forensic application for the Guanzhong Han population. The population comparison showed the Guanzhong Han had a close genetic relationship with the Ningxia Han, Tujia and Bai populations among the populations tested.

## Author Contributions

B.Z. and Y.Z. wrote the main manuscript text, X.T., H.M., W.L., H.W., G.Y., R.J. and C.Y did the data processing and the manuscript modification, and J.Y. and C.S. prepared the figures. All authors reviewed the manuscript.

## Supplementary Material

Supplementary Information supplementary information

## Figures and Tables

**Table 1:** The allelic frequencies and statistical parameters for the 21 STR loci in Han population from Guanzhong region, Shaanxi, China (n = 275)

Allele	D6S474	D12ATA63	D22S1045	D10S1248	D1S1677	D11S4463	D1S1627	D3S4529	D2S441	D6S1017	D4S2408	D19S433	D17S1301	D1GATA113	D18S853	D20S482	D14S1434	D9S1122	D2S1776	D10S1435	D5S2500
7										0.0036				0.5145					0.0018		
8										0.2236	0.2364		0.0055	0.0091						0.0236	
9						0.0036					0.2945		0.0236			0.0018		0.0036	0.1200	0.0036	
9.1									0.0145												
10					0.0091		0.0291		0.2309	0.3582	0.2964		0.0655	0.0036	0.0091	0.0345	0.1073	0.0782	0.0673	0.0418	
10.1									0.0109												
10.3																				0.0018	
11		0.0018	0.2436	0.0109		0.0018	0.0127		0.3473	0.0364	0.1545	0.0036	0.1782	0.1691	0.4036	0.0109	0.1636	0.1709	0.2818	0.1618	
11.3									0.0309												
12		0.3327	0.0018	0.0673	0.0182	0.0509	0.0982	0.0018	0.2109	0.2818	0.0182	0.0418	0.4582	0.2782	0.0582	0.0673	0.0218	0.3182	0.3800	0.3473	
12.2												0.0055									
12.3													0.0018								
13	0.0018	0.0036	0.0091	0.3545	0.0891	0.2073	0.5564	0.1891	0.0200	0.0891		0.2836	0.2109	0.0255	0.2436	0.2382	0.3091	0.3509	0.1164	0.2745	
13.2												0.0564									
14	0.3745	0.0382	0.0109	0.2636	0.5309	0.3236	0.2927	0.2055	0.1200	0.0073		0.2545	0.0509		0.2309	0.3873	0.3709	0.0709	0.0327	0.1291	0.4073
14.1									0.0018												
14.2												0.1055									
15	0.3636	0.0018	0.2855	0.2200	0.3073	0.2855	0.0091	0.4182	0.0127			0.0709	0.0055		0.0545	0.1855	0.0236	0.0036		0.0145	
15.2												0.1345									
16	0.1345	0.1600	0.2582	0.0691	0.0418	0.0964	0.0018	0.1564				0.0182				0.0745	0.0036	0.0036		0.0018	
16.2												0.0218									
17	0.0909	0.3545	0.1655	0.0127	0.0036	0.0273		0.0291				0.0018									0.2873
17.2												0.0018									
18	0.0327	0.0927	0.0218	0.0018		0.0036															0.2327
19	0.0018	0.0109	0.0036																		0.0018
20		0.0036																			0.0491
23																					0.0200
24																					0.0018
PD	0.8576	0.8858	0.8979	0.8906	0.7898	0.9034	0.7700	0.8775	0.9072	0.8828	0.8863	0.9437	0.8753	0.8091	0.8566	0.8975	0.8825	0.8852	0.8866	0.8969	0.8452
PIC	0.6476	0.6825	0.7243	0.7057	0.5527	0.7192	0.5325	0.6787	0.7299	0.6870	0.6994	0.7916	0.6672	0.5674	0.6713	0.7107	0.6822	0.6915	0.7040	0.7216	0.6386
PE	0.4199	0.4034	0.5790	0.5144	0.2822	0.4540	0.2865	0.4255	0.5082	0.4599	0.4717	0.5856	0.4540	0.2738	0.5462	0.5144	0.4658	0.4599	0.5207	0.5790	0.4599
TPI	1.6369	1.5805	2.3707	2.0221	1.2277	1.7628	1.2387	1.6566	1.9928	1.7857	1.8333	2.4123	1.7628	1.2061	2.1825	2.0221	1.8092	1.7857	2.0522	2.3707	1.7857
HO	0.6945	0.6836	0.7891	0.7527	0.5927	0.7164	0.5964	0.6982	0.7491	0.7200	0.7273	0.7927	0.7164	0.5855	0.7709	0.7527	0.7236	0.7200	0.7564	0.7891	0.7200
HE	0.7000	0.7278	0.7644	0.7468	0.6136	0.7582	0.5940	0.7219	0.7653	0.7329	0.7453	0.8147	0.7063	0.6285	0.7180	0.7475	0.7276	0.7352	0.7426	0.7586	0.6946
*p*	0.8062	0.0898	0.3622	0.8618	0.4535	0.0938	0.9664	0.3543	0.4888	0.5919	0.4591	0.3147	0.7501	0.1289	0.0567	0.8824	0.8448	0.5327	0.6383	0.2590	0.3848

PD: power of discrimination, PIC: polymorphism information content, PE: probability of exclusion, TPI: typical paternity index, HO: observed heterozygosity, HE: expected heterozygosity, *p*: probability values of exact tests for Hardy-Weinberg equilibrium.

**Table 2:** Pairwise *Fst* and associated *p* values of 21 STR loci between Chinese Guanzhong Han population and 10 reference populations

Population	index	D10S1248	D10S1435	D11S4463	D12ATA63	D14S1434	D17S1301	D18S853	D19S433	D1GATA113	D1S1677	D20S482	D22S1045	D2S1776	D2S441	D3S4529	D4S2408	D5S2500	D6S1017	D6S474	D9S1122	D1S1627
Bai	*Fst*	−0.0018	−0.0027	−0.0028	−0.0010	0.0004	0.0008	0.0061	−0.0023	0.0034	0.0159	0.0015	0.0020	−0.0020	0.0009	0.0028	0.0024	−0.0004	0.0064	−0.0004	0.0013	−0.0005
	*p*	1.0000	1.0000	1.0000	0.9306	0.6285	0.5093	0.0684	1.0000	0.2102	0.0029	0.4115	0.3392	1.0000	0.5200	0.2590	0.3060	0.7478	0.0635	0.7820	0.4614	0.7586
Kazak	*Fst*	0.0017	0.0016	0.0014	0.0105	−0.0024	0.0131	0.0019	0.0033	0.0165	0.0114	0.0002	0.0032	0.0034	0.0086	0.0080	0.0083	0.0209	−0.0001	0.0004	0.0017	0.0066
	*p*	0.3353	0.3617	0.4135	0.0108	1.0000	0.0020	0.3148	0.1417	0.0049	0.0117	0.6579	0.1789	0.1613	0.0166	0.0313	0.0244	0.0010	0.7243	0.5943	0.3441	0.0557
Ningxia Han	*Fst*	−0.0006	0.0002	0.0008	−0.0019	0.0010	−0.0009	−0.0005	−0.0008	0.0001	−0.0008	0.0011	0.0001	−0.0009	−0.0008	0.0007	0.0028	−0.0020	0.0013	0.0012	0.0010	−0.0012
	*p*	0.9443	0.6452	0.4800	1.0000	0.3998	0.9834	0.9081	0.9961	0.5904	0.9414	0.3382	0.6833	0.9980	0.9853	0.4741	0.1193	1.0000	0.3363	0.3920	0.4135	1.0000
Russian	*Fst*	−0.0026	0.0005	0.0036	0.0041	0.0036	−0.0025	−0.0018	−0.0007	0.0042	0.0000	−0.0008	0.0881	−0.0016	0.0020	0.0078	0.0002	0.0006	0.0023	0.0070	−0.0023	0.0010
	*p*	1.0000	0.5992	0.1867	0.1232	0.1730	1.0000	1.0000	0.9433	0.1623	0.6002	0.9179	0.0000	1.0000	0.3148	0.0332	0.6804	0.5376	0.2972	0.0479	1.0000	0.4262
Salar	*Fst*	0.0036	0.0003	0.0015	−0.0001	−0.0008	0.0015	−0.0010	−0.0019	0.0049	0.0046	0.0002	0.0700	0.0033	0.0053	0.0110	0.0014	0.0042	−0.0002	0.0025	0.0021	−0.0020
	*p*	0.1320	0.6637	0.4135	0.7126	0.9130	0.3529	0.9521	1.0000	0.1202	0.1222	0.6520	0.0000	0.1652	0.0733	0.0049	0.4242	0.1320	0.7722	0.2405	0.2991	1.0000
Tibetan	*Fst*	0.0056	0.0038	0.0006	0.0216	0.0061	0.0034	0.0046	0.0032	0.0002	0.0009	0.0039	0.0162	−0.0013	0.0082	0.0202	0.0048	0.0004	0.0103	−0.0031	0.0007	−0.0001
	*p*	0.0772	0.1642	0.5748	0.0000	0.0763	0.1975	0.1193	0.1887	0.5904	0.4555	0.1633	0.0000	0.9814	0.0244	0.0000	0.1300	0.5552	0.0127	1.0000	0.5601	0.6393
Tujia	*Fst*	−0.0028	−0.0021	−0.0022	0.0005	0.0138	−0.0002	−0.0002	−0.0010	−0.0017	0.0019	0.0066	−0.0002	−0.0020	0.0100	0.0017	0.0002	−0.0021	0.0015	0.0043	−0.0010	−0.0007
	*p*	1.0000	1.0000	1.0000	0.5894	0.0068	0.7615	0.7488	0.9873	0.9980	0.3314	0.0469	0.7634	1.0000	0.0108	0.3842	0.6373	1.0000	0.4106	0.1505	0.9472	0.7859
Uigur	*Fst*	0.0100	0.0026	0.0056	0.0184	0.0025	0.0022	0.0022	0.0034	0.0229	0.0221	0.0009	0.0034	0.0020	0.0076	0.0132	0.0038	0.0178	0.0059	0.0116	0.0006	0.0243
	*p*	0.0010	0.1232	0.0137	0.0000	0.1535	0.1906	0.1711	0.0469	0.0000	0.0000	0.4233	0.0772	0.1857	0.0020	0.0000	0.0675	0.0000	0.0205	0.0010	0.4663	0.0000
Yi	*Fst*	−0.0022	0.0227	0.0019	0.0079	0.0131	0.0120	0.0002	0.0003	0.0419	−0.0026	0.0068	0.0880	0.0030	0.0070	0.0049	0.0146	−0.0005	0.0024	0.0009	0.0042	0.0005
	*p*	1.0000	0.0000	0.3431	0.0362	0.0029	0.0098	0.6804	0.7302	0.0000	1.0000	0.0401	0.0000	0.2180	0.0362	0.1193	0.0029	0.7869	0.2454	0.4712	0.1564	0.5484
Mongolian	*Fst*	−0.0012	0.0001	0.0011	0.0093	0.0034	0.0033	0.0026	−0.0017	0.0062	0.0070	−0.0022	0.0075	0.0003	0.0108	−0.0017	0.0076	0.0089	0.0001	−0.0027	−0.0036	−0.0026
	*p*	0.9414	0.7498	0.5112	0.0411	0.2287	0.2268	0.2669	1.0000	0.1359	0.0958	1.0000	0.0401	0.6676	0.0225	0.9902	0.0694	0.0538	0.6960	1.0000	1.0000	1.0000

**Table 3:** The *D_A_* distances between Guanzhong Han population and other groups based on 10 STR loci

Index	Ningxia Han	Tujia	Bai	Kazak	Tibetan	Mongolian	Uigur	Salar	Russian	Yi
*D_A_*	0.0073	0.0077	0.0091	0.0126	0.0133	0.0141	0.0153	0.0264	0.0281	0.0337
