# Supplementary material for: Genetic Variability and Phylogenetic Analysis of Han Population from Guanzhong Region of China based on 21 non-CODIS STR Loci
Source: Sci Rep. 2015 Mar 9;5:8872. doi: 10.1038/srep08872 (PMC4352849; doi:10.1038/srep08872)
Supplement: Supplementary Information [file srep08872-s1.doc]

**Genetic Variability and Phylogenetic Analysis of Han Population from Guanzhong Region of China based on 21 non-CODIS STR Loci**

**Author:**

**Yu-Dang Zhang^1,2,*^, Xiao-Li Tang^3,*^, Hao-Tian Meng^1,2^, Hong-Dan Wang^4^, Rui Jin^2,5^, Chun-Hua Yang^2,6^, Jiang-Wei Yan^7^, Guang Yang^8^, Wen-Juan Liu^2^, Chun-Mei Shen^9^, Bo-Feng Zhu^1^**

*^1^Research Center of Stomatology, Stomatological Hospital, Xi'an Jiaotong University**, Xi'an, 710004, P. R. China*

*^2^Xi’an Jiaotong University Health Science Center, Xi’an 710061, P. R. China*

*^3^Department of Biochemistry, Medical college of Nanchang University, Nanchang, 330001, P. R. China*

*^4^Medical Genetic Institute of Henan Province, Henan Provincial People's Hospital, People's Hospital of Zhengzhou University, Zhengzhou 450003, P. R. China*

*^5^Department of Radiology,The Second Affiliated Hospital of Xi'an Jiaotong University, Xi'an, 710004, P. R. China*

*^6^People’s hospital of Arong Banner, Hulun Buir City, 162750. P. R. China.*

*^7^Key Laboratory of Genome Sciences, Beijing Institute of Genomics, Chinese Academy of Sciences, Beijing 100101, P. R. China*

^8^*Department of Pathology and Laboratory Medicine Loma Linda University Medical Center, California, 92354, USA*

^9^*College of Life Sciences, Shaanxi Normal University, Xi'an, 710062, P. R. China*

**1, Yu-Dang Zhang**

Research Center of Stomatology, Stomatological Hospital, Xi'an Jiaotong University, Xi'an, 710004, P. R. China

Xi’an Jiaotong University Health Science Center, Xi’an 710061, P. R. China

**2. Xiao-Li Tang**

Department of Biochemistry, Medical college of Nanchang University, Nanchang, 330001, P. R. China

**3, Hao-Tian Meng**

Research Center of Stomatology, Stomatological Hospital, Xi'an Jiaotong University, Xi'an, 710004, P. R. China

Xi’an Jiaotong University Health Science Center, Xi’an 710061, P. R. China

4**, Hong-Dan Wang**

Medical Genetic Institute of Henan Province, Henan Provincial People's Hospital, People's Hospital of Zhengzhou University, Zhengzhou 450003, P. R. China

**5, Rui Jin**

Xi’an Jiaotong University Health Science Center, Xi’an 710061, P. R. China

Department of Radiology, The Second Affiliated Hospital of Xi'an Jiaotong University, Xi'an, 710004, P. R. China

**6, Chun-Hua Yang**

Xi’an Jiaotong University Health Science Center, Xi’an 710061, P. R. China

People’s hospital of Arong Banner, Hulun Buir City 162750, P. R. China.

**7, Jiang-Wei Yan**

Key Laboratory of Genome Sciences, Beijing Institute of Genomics, Chinese Academy of Sciences, Beijing 100101, P. R. China

**8, Guang Yang**

Department of Pathology and Laboratory Medicine Loma Linda University Medical Center, California 92354, USA

**9, Wen-Juan Liu**

Xi’an Jiaotong University Health Science Center, Xi’an 710061, P. R. China

**10, Chun-Mei Shen**

College of Life Sciences, Shaanxi Normal University, Xi'an, 710062, P. R. China

**11, Bo-Feng Zhu**

Research Center of Stomatology, Stomatological Hospital, Xi'an Jiaotong University, Xi’an 710004, P. R. China

**Correspondence:** Prof. Bo-feng Zhu, Research Center of Stomatology, Stomatological Hospital, Xi'an Jiaotong University, Xi’an 710004, P. R. China

E-mail: [zhubofeng7372@126.com](mailto:zhubofeng7372@126.com)

Tel.: +86-029-82655116

*Both authors contributed equally to this work.

**Legend of Figure:**

Figure -1. The geographical locations of the Guanzhong Han and 10 reference groups in China. The map was created in matlab R2013b software (MathWorks Inc., USA).

Figure-2. Phylogenetic tree for Guanzhong Han and 10 reference populations constructed by the software MEGA v5 based on *D_A_* distances (A) and by the software PHYLIP v3.6 based on allelic frequency (B), respectively.

Figure-3. Principal component analysis plot structured based on allelic frequencies of 21 STR loci in 11 populations.

| Sample Name  Supplementary Table 1 The genotyping results of the 21 STR loci from Guanzhong Han, Shaanxi, China (n = 275)   |  |  |  |  |  |  |  |  |  |  |  |  |  |  |  |  |  |  |  |  |  |  |  |  |  |  |  |  |  |  |  |  |  |  |  |  |  |  |  |  |  |  |  | | --- | --- | --- | --- | --- | --- | --- | --- | --- | --- | --- | --- | --- | --- | --- | --- | --- | --- | --- | --- | --- | --- | --- | --- | --- | --- | --- | --- | --- | --- | --- | --- | --- | --- | --- | --- | --- | --- | --- | --- | --- | --- | --- | | D6S474 | D6S474 | D12ATA63 | D12ATA63 | D22S1045 | D22S1045 | D10S1248 | D10S1248 | D1S1677 | D1S1677 | D11S4463 | D11S4463 | D1S1627 | D1S1627 | D3S4529 | D3S4529 | D2S441 | D2S441 | D6S1017 | D6S1017 | D4S2408 | D4S2408 | D19S433 | D19S433 | D17S1301 | D17S1301 | D1GATA113 | D1GATA113 | D18S853 | D18S853 | D20S482 | D20S482 | D14S1434 | D14S1434 | D9S1122 | D9S1122 | D2S1776 | D2S1776 | D10S1435 | D10S1435 | D5S2500 | D5S2500 |
| --- | --- | --- | --- | --- | --- | --- | --- | --- | --- | --- | --- | --- | --- | --- | --- | --- | --- | --- | --- | --- | --- | --- | --- | --- | --- | --- | --- | --- | --- | --- | --- | --- | --- | --- | --- | --- | --- | --- | --- | --- | --- | --- | --- | --- | --- | --- | --- | --- | --- | --- | --- | --- | --- | --- | --- | --- | --- | --- | --- | --- | --- | --- | --- | --- | --- | --- | --- | --- | --- | --- | --- | --- | --- | --- | --- | --- | --- | --- | --- | --- | --- | --- | --- | --- | --- |
| 1 | 14 | 16 | 17 | 17 | 16 | 17 | 12 | 13 | 14 | 15 | 13 | 14 | 13 | 14 | 16 | 16 | 12 | 14 | 8 | 12 | 9 | 11 | 13 | 14.2 | 10 | 14 | 7 | 12 | 12 | 13 | 14 | 15 | 12 | 13 | 12 | 13 | 10 | 12 | 11 | 13 | 14 | 14 |
| 2 | 14 | 15 | 12 | 17 | 11 | 17 | 14 | 16 | 14 | 14 | 13 | 13 | 13 | 13 | 15 | 15 | 10 | 12 | 8 | 14 | 9 | 10 | 14 | 16 | 12 | 13 | 7 | 7 | 11 | 12 | 13 | 15 | 13 | 14 | 11 | 13 | 11 | 13 | 12 | 12 | 14 | 17 |
| 3 | 15 | 16 | 12 | 12 | 11 | 18 | 13 | 13 | 14 | 14 | 14 | 15 | 13 | 14 | 14 | 14 | 12 | 12 | 12 | 12 | 8 | 11 | 15.2 | 16.2 | 12 | 12 | 12 | 12 | 11 | 11 | 11 | 13 | 13 | 13 | 12 | 14 | 11 | 12 | 10 | 12 | 14 | 19 |
| 4 | 14 | 15 | 12 | 17 | 11 | 15 | 12 | 14 | 13 | 16 | 15 | 15 | 10 | 13 | 16 | 16 | 14 | 14 | 12 | 12 | 9 | 9 | 13 | 14.2 | 12 | 13 | 7 | 7 | 10 | 11 | 14 | 15 | 11 | 13 | 11 | 12 | 9 | 9 | 12 | 14 | 17 | 18 |
| 5 | 15 | 16 | 17 | 17 | 16 | 17 | 13 | 15 | 14 | 15 | 15 | 15 | 13 | 14 | 14 | 15 | 10 | 11 | 8 | 10 | 10 | 11 | 16 | 16.2 | 12 | 14 | 7 | 7 | 13 | 14 | 13 | 15 | 12 | 14 | 12 | 13 | 12 | 13 | 12 | 13 | 14 | 17 |
| 6 | 14 | 18 | 16 | 16 | 13 | 15 | 13 | 15 | 14 | 15 | 14 | 14 | 13 | 13 | 15 | 16 | 12 | 14 | 12 | 13 | 8 | 11 | 13 | 14 | 12 | 12 | 7 | 12 | 11 | 13 | 14 | 15 | 14 | 14 | 11 | 12 | 11 | 13 | 12 | 14 | 14 | 18 |
| 7 | 15 | 16 | 17 | 18 | 11 | 14 | 13 | 14 | 14 | 15 | 13 | 14 | 12 | 13 | 13 | 15 | 10 | 14 | 8 | 8 | 9 | 11 | 15.2 | 15.2 | 11 | 12 | 7 | 11 | 12 | 14 | 13 | 14 | 10 | 14 | 13 | 13 | 9 | 9 | 13 | 14 | 17 | 18 |
| 8 | 14 | 14 | 12 | 12 | 16 | 16 | 13 | 14 | 14 | 14 | 13 | 16 | 13 | 13 | 15 | 15 | 11 | 11 | 10 | 10 | 9 | 9 | 13 | 14.2 | 11 | 12 | 7 | 13 | 11 | 12 | 13 | 14 | 13 | 14 | 11 | 13 | 9 | 9 | 12 | 14 | 14 | 17 |
| 9 | 15 | 15 | 16 | 17 | 15 | 18 | 14 | 15 | 15 | 15 | 15 | 16 | 14 | 14 | 15 | 15 | 11.3 | 12 | 8 | 10 | 8 | 9 | 13 | 13 | 8 | 12 | 7 | 12 | 11 | 13 | 13 | 14 | 11 | 14 | 11 | 12 | 11 | 12 | 12 | 13 | 17 | 20 |
| 10 | 14 | 15 | 17 | 17 | 15 | 16 | 14 | 15 | 14 | 14 | 14 | 14 | 13 | 14 | 14 | 15 | 9.1 | 12 | 10 | 13 | 9 | 10 | 14.2 | 15.2 | 11 | 12 | 7 | 12 | 11 | 11 | 10 | 14 | 11 | 14 | 12 | 13 | 12 | 14 | 11 | 14 | 14 | 17 |
| 11 | 15 | 17 | 12 | 12 | 16 | 17 | 14 | 15 | 14 | 14 | 15 | 15 | 13 | 14 | 13 | 13 | 11 | 12 | 10 | 10 | 9 | 10 | 13 | 14.2 | 12 | 13 | 7 | 7 | 11 | 14 | 13 | 16 | 10 | 14 | 12 | 13 | 10 | 12 | 13 | 14 | 14 | 24 |
| 12 | 14 | 15 | 16 | 18 | 13 | 17 | 13 | 17 | 14 | 15 | 13 | 14 | 13 | 14 | 17 | 17 | 10 | 11 | 8 | 12 | 9 | 10 | 14.2 | 15.2 | 11 | 11 | 7 | 12 | 11 | 11 | 13 | 16 | 13 | 14 | 12 | 13 | 11 | 13 | 12 | 13 | 17 | 18 |
| 13 | 14 | 17 | 17 | 18 | 11 | 17 | 13 | 15 | 12 | 14 | 13 | 13 | 12 | 13 | 15 | 16 | 10 | 14 | 8 | 12 | 10 | 10 | 13.2 | 15 | 12 | 12 | 12 | 12 | 10 | 11 | 13 | 14 | 11 | 13 | 11 | 13 | 9 | 11 | 13 | 13 | 17 | 17 |
| 14 | 14 | 15 | 12 | 12 | 15 | 17 | 14 | 16 | 15 | 15 | 13 | 15 | 12 | 13 | 13 | 15 | 11 | 11 | 10 | 10 | 10 | 10 | 13 | 13 | 11 | 12 | 7 | 7 | 11 | 13 | 13 | 15 | 11 | 14 | 10 | 12 | 11 | 12 | 10 | 13 | 14 | 18 |
| 15 | 14 | 17 | 12 | 17 | 16 | 16 | 13 | 14 | 13 | 14 | 14 | 15 | 13 | 14 | 13 | 13 | 11 | 13 | 10 | 12 | 8 | 10 | 13 | 13 | 12 | 12 | 7 | 11 | 11 | 14 | 14 | 14 | 14 | 14 | 11 | 13 | 12 | 13 | 13 | 14 | 14 | 17 |
| 16 | 14 | 14 | 17 | 17 | 15 | 16 | 14 | 16 | 14 | 14 | 15 | 16 | 12 | 12 | 13 | 14 | 10 | 10 | 8 | 13 | 8 | 10 | 14 | 14.2 | 12 | 13 | 7 | 12 | 11 | 13 | 13 | 14 | 11 | 14 | 12 | 13 | 11 | 13 | 13 | 13 | 18 | 23 |
| 17 | 14 | 16 | 15 | 17 | 15 | 16 | 12 | 15 | 14 | 15 | 13 | 14 | 13 | 14 | 15 | 15 | 11 | 12 | 12 | 12 | 10 | 11 | 13 | 14 | 12 | 13 | 7 | 12 | 13 | 15 | 10 | 12 | 14 | 14 | 12 | 13 | 12 | 13 | 12 | 14 | 14 | 18 |
| 18 | 15 | 15 | 12 | 12 | 11 | 11 | 13 | 13 | 14 | 14 | 14 | 14 | 13 | 14 | 14 | 15 | 10 | 14 | 12 | 13 | 8 | 9 | 13 | 15 | 11 | 12.3 | 7 | 11 | 13 | 15 | 12 | 14 | 11 | 13 | 12 | 12 | 9 | 12 | 12 | 13 | 17 | 18 |
| 19 | 14 | 15 | 12 | 12 | 11 | 16 | 13 | 15 | 13 | 14 | 13 | 15 | 13 | 14 | 15 | 16 | 12 | 12 | 12 | 13 | 8 | 11 | 13 | 14 | 11 | 12 | 8 | 11 | 13 | 14 | 10 | 14 | 11 | 14 | 12 | 12 | 12 | 12 | 11 | 13 | 14 | 17 |
| 20 | 14 | 15 | 12 | 18 | 15 | 15 | 14 | 14 | 14 | 15 | 13 | 14 | 13 | 14 | 15 | 16 | 10 | 11 | 11 | 12 | 10 | 10 | 13 | 14 | 12 | 12 | 7 | 12 | 11 | 15 | 13 | 14 | 13 | 14 | 13 | 13 | 12 | 12 | 11 | 12 | 14 | 17 |
| 21 | 14 | 15 | 12 | 17 | 11 | 11 | 14 | 15 | 14 | 15 | 13 | 15 | 13 | 13 | 13 | 14 | 10 | 14 | 8 | 13 | 9 | 11 | 13 | 14 | 11 | 12 | 7 | 7 | 13 | 15 | 10 | 14 | 14 | 14 | 10 | 12 | 10 | 12 | 10 | 12 | 14 | 17 |
| 22 | 15 | 15 | 12 | 17 | 16 | 16 | 14 | 16 | 14 | 14 | 12 | 13 | 13 | 13 | 14 | 16 | 11.3 | 12 | 10 | 10 | 10 | 11 | 13 | 15.2 | 12 | 12 | 7 | 12 | 11 | 11 | 14 | 15 | 13 | 14 | 12 | 12 | 9 | 11 | 8 | 13 | 14 | 18 |
| 23 | 16 | 17 | 12 | 17 | 11 | 15 | 15 | 15 | 14 | 14 | 9 | 13 | 13 | 14 | 13 | 15 | 11 | 11 | 8 | 12 | 9 | 10 | 14 | 14.2 | 11 | 12 | 7 | 12 | 11 | 13 | 13 | 14 | 10 | 10 | 11 | 13 | 12 | 12 | 13 | 14 | 14 | 18 |
| 24 | 15 | 16 | 12 | 17 | 16 | 16 | 13 | 14 | 14 | 15 | 14 | 15 | 13 | 14 | 14 | 16 | 11 | 11.3 | 12 | 13 | 8 | 9 | 14 | 15.2 | 11 | 13 | 7 | 7 | 11 | 13 | 14 | 16 | 14 | 15 | 10 | 14 | 12 | 12 | 12 | 13 | 17 | 17 |
| 25 | 14 | 15 | 14 | 17 | 11 | 11 | 13 | 13 | 14 | 16 | 15 | 16 | 13 | 15 | 13 | 15 | 12 | 14 | 10 | 10 | 9 | 10 | 14 | 15.2 | 13 | 13 | 7 | 11 | 13 | 14 | 16 | 16 | 13 | 14 | 13 | 13 | 11 | 14 | 11 | 14 | 14 | 14 |
| 26 | 14 | 15 | 12 | 17 | 15 | 17 | 14 | 14 | 14 | 14 | 13 | 15 | 13 | 13 | 14 | 14 | 12 | 14 | 10 | 12 | 9 | 11 | 13 | 13 | 12 | 13 | 7 | 12 | 11 | 13 | 14 | 16 | 13 | 14 | 12 | 13 | 12 | 13 | 11 | 13 | 14 | 17 |
| 27 | 15 | 16 | 12 | 16 | 15 | 16 | 13 | 14 | 13 | 14 | 14 | 15 | 13 | 14 | 14 | 16 | 10 | 11 | 10 | 12 | 9 | 9 | 12 | 13 | 11 | 12 | 7 | 7 | 11 | 11 | 14 | 16 | 10 | 14 | 10 | 12 | 12 | 13 | 12 | 14 | 14 | 17 |
| 28 | 14 | 15 | 17 | 17 | 15 | 16 | 12 | 13 | 14 | 15 | 15 | 15 | 13 | 14 | 14 | 16 | 11 | 14 | 8 | 12 | 9 | 11 | 13 | 14 | 12 | 13 | 7 | 11 | 11 | 11 | 14 | 15 | 10 | 14 | 11 | 11 | 10 | 10 | 11 | 14 | 14 | 18 |
| 29 | 14 | 15 | 12 | 12 | 15 | 16 | 13 | 16 | 13 | 14 | 14 | 15 | 12 | 13 | 14 | 14 | 10 | 12 | 8 | 10 | 10 | 11 | 13.2 | 14 | 13 | 13 | 7 | 11 | 11 | 14 | 15 | 16 | 13 | 14 | 12 | 14 | 10 | 11 | 12 | 14 | 14 | 14 |
| 30 | 14 | 15 | 12 | 17 | 16 | 17 | 14 | 16 | 10 | 14 | 13 | 14 | 13 | 13 | 15 | 16 | 10 | 12 | 12 | 12 | 8 | 10 | 13 | 13.2 | 12 | 12 | 7 | 7 | 11 | 13 | 15 | 15 | 13 | 14 | 11 | 14 | 12 | 12 | 11 | 13 | 18 | 18 |
| 31 | 14 | 19 | 12 | 16 | 15 | 16 | 13 | 14 | 14 | 14 | 15 | 15 | 14 | 14 | 13 | 16 | 11 | 11 | 8 | 10 | 9 | 10 | 13 | 15.2 | 11 | 12 | 7 | 7 | 13 | 14 | 14 | 15 | 14 | 14 | 11 | 12 | 11 | 12 | 13 | 13 | 14 | 17 |
| 32 | 14 | 17 | 16 | 16 | 16 | 16 | 13 | 13 | 14 | 14 | 14 | 16 | 13 | 14 | 13 | 16 | 11 | 12 | 10 | 10 | 9 | 12 | 13.2 | 14.2 | 9 | 11 | 7 | 12 | 11 | 13 | 13 | 14 | 13 | 14 | 11 | 12 | 11 | 11 | 11 | 12 | 17 | 18 |
| 33 | 15 | 15 | 16 | 16 | 16 | 17 | 14 | 15 | 14 | 14 | 14 | 15 | 13 | 14 | 13 | 16 | 10 | 11 | 8 | 10 | 10 | 11 | 12.2 | 13 | 12 | 14 | 12 | 12 | 11 | 13 | 14 | 15 | 13 | 14 | 13 | 14 | 9 | 11 | 11 | 13 | 14 | 17 |
| 34 | 15 | 18 | 17 | 17 | 11 | 15 | 13 | 14 | 14 | 14 | 13 | 14 | 13 | 14 | 13 | 15 | 12 | 14 | 10 | 12 | 8 | 9 | 15.2 | 15.2 | 12 | 13 | 12 | 12 | 11 | 14 | 12 | 14 | 12 | 13 | 12 | 14 | 11 | 12 | 12 | 13 | 14 | 14 |
| 35 | 15 | 16 | 12 | 12 | 11 | 15 | 13 | 15 | 13 | 13 | 15 | 15 | 13 | 13 | 14 | 15 | 10 | 11 | 10 | 13 | 8 | 9 | 14 | 14 | 10 | 13 | 7 | 11 | 11 | 14 | 13 | 13 | 14 | 14 | 11 | 13 | 9 | 12 | 12 | 13 | 14 | 17 |
| 36 | 14 | 15 | 16 | 17 | 11 | 17 | 15 | 16 | 14 | 14 | 13 | 15 | 13 | 13 | 13 | 15 | 10.1 | 11 | 8 | 12 | 8 | 9 | 13 | 14 | 11 | 13 | 11 | 12 | 12 | 13 | 13 | 13 | 10 | 14 | 13 | 13 | 11 | 11 | 11 | 12 | 17 | 18 |
| 37 | 14 | 15 | 12 | 16 | 11 | 11 | 13 | 13 | 14 | 14 | 13 | 14 | 13 | 14 | 13 | 15 | 10 | 11 | 12 | 12 | 8 | 9 | 12 | 15.2 | 11 | 12 | 7 | 12 | 11 | 12 | 14 | 14 | 14 | 15 | 11 | 12 | 9 | 12 | 8 | 13 | 17 | 18 |
| 38 | 14 | 16 | 12 | 12 | 11 | 11 | 14 | 16 | 14 | 14 | 13 | 16 | 12 | 13 | 13 | 14 | 11.3 | 14 | 10 | 10 | 10 | 11 | 13 | 15.2 | 12 | 12 | 12 | 12 | 11 | 11 | 13 | 13 | 13 | 14 | 12 | 13 | 11 | 12 | 13 | 14 | 14 | 20 |
| 39 | 15 | 16 | 12 | 17 | 16 | 19 | 13 | 15 | 14 | 14 | 14 | 14 | 12 | 15 | 14 | 15 | 11 | 14 | 10 | 10 | 8 | 10 | 12 | 14.2 | 12 | 12 | 7 | 11 | 14 | 15 | 12 | 13 | 11 | 13 | 12 | 13 | 11 | 13 | 12 | 12 | 14 | 17 |
| 40 | 15 | 15 | 16 | 18 | 11 | 16 | 13 | 16 | 14 | 15 | 12 | 14 | 14 | 14 | 14 | 15 | 10 | 12 | 11 | 13 | 8 | 9 | 14 | 15 | 12 | 12 | 7 | 11 | 12 | 12 | 13 | 14 | 13 | 14 | 11 | 12 | 12 | 13 | 10 | 11 | 14 | 18 |
| 41 | 14 | 16 | 16 | 18 | 11 | 16 | 11 | 13 | 10 | 14 | 15 | 15 | 13 | 14 | 13 | 14 | 10 | 10 | 8 | 13 | 8 | 9 | 13 | 15 | 9 | 11 | 7 | 12 | 11 | 13 | 9 | 14 | 13 | 13 | 12 | 14 | 11 | 11 | 12 | 13 | 14 | 14 |
| Sample Name  Supplementary Table 1 (*Continued*)   |  |  |  |  |  |  |  |  |  |  |  |  |  |  |  |  |  |  |  |  |  |  |  |  |  |  |  |  |  |  |  |  |  |  |  |  |  |  |  |  |  |  |  | | --- | --- | --- | --- | --- | --- | --- | --- | --- | --- | --- | --- | --- | --- | --- | --- | --- | --- | --- | --- | --- | --- | --- | --- | --- | --- | --- | --- | --- | --- | --- | --- | --- | --- | --- | --- | --- | --- | --- | --- | --- | --- | --- | | D6S474 | D6S474 | D12ATA63 | D12ATA63 | D22S1045 | D22S1045 | D10S1248 | D10S1248 | D1S1677 | D1S1677 | D11S4463 | D11S4463 | D1S1627 | D1S1627 | D3S4529 | D3S4529 | D2S441 | D2S441 | D6S1017 | D6S1017 | D4S2408 | D4S2408 | D19S433 | D19S433 | D17S1301 | D17S1301 | D1GATA113 | D1GATA113 | D18S853 | D18S853 | D20S482 | D20S482 | D14S1434 | D14S1434 | D9S1122 | D9S1122 | D2S1776 | D2S1776 | D10S1435 | D10S1435 | D5S2500 | D5S2500 |
| 42 | 14 | 16 | 16 | 18 | 15 | 17 | 14 | 15 | 12 | 14 | 14 | 15 | 12 | 13 | 16 | 16 | 10 | 12 | 10 | 10 | 8 | 9 | 14 | 14 | 10 | 11 | 7 | 12 | 11 | 15 | 13 | 15 | 10 | 13 | 12 | 13 | 11 | 12 | 11 | 14 | 14 | 17 |
| 43 | 14 | 15 | 12 | 17 | 15 | 17 | 14 | 15 | 14 | 15 | 15 | 15 | 13 | 14 | 13 | 15 | 10 | 11 | 8 | 12 | 8 | 9 | 13 | 14.2 | 12 | 12 | 12 | 12 | 11 | 11 | 13 | 14 | 10 | 14 | 12 | 14 | 11 | 13 | 13 | 13 | 14 | 18 |
| 44 | 14 | 14 | 17 | 17 | 11 | 15 | 13 | 14 | 14 | 15 | 14 | 14 | 13 | 14 | 13 | 14 | 10 | 10 | 8 | 10 | 10 | 10 | 13 | 14.2 | 12 | 13 | 12 | 12 | 12 | 13 | 15 | 15 | 11 | 13 | 10 | 10 | 10 | 12 | 8 | 12 | 14 | 20 |
| 45 | 14 | 16 | 17 | 18 | 16 | 16 | 14 | 15 | 14 | 14 | 14 | 14 | 13 | 13 | 15 | 16 | 11 | 12 | 8 | 8 | 9 | 10 | 12 | 12 | 11 | 12 | 7 | 11 | 12 | 13 | 13 | 13 | 10 | 11 | 10 | 13 | 11 | 12 | 13 | 14 | 14 | 14 |
| 46 | 14 | 16 | 17 | 17 | 11 | 11 | 15 | 18 | 14 | 15 | 14 | 15 | 10 | 13 | 13 | 16 | 11 | 12 | 12 | 12 | 9 | 10 | 11 | 15.2 | 11 | 13 | 12 | 12 | 11 | 11 | 14 | 15 | 13 | 14 | 10 | 13 | 9 | 14 | 12 | 14 | 14 | 17 |
| 47 | 16 | 17 | 14 | 16 | 15 | 17 | 14 | 14 | 13 | 15 | 15 | 16 | 13 | 14 | 14 | 15 | 11 | 14 | 8 | 10 | 8 | 9 | 14.2 | 16.2 | 9 | 12 | 11 | 12 | 14 | 15 | 13 | 15 | 11 | 12 | 13 | 13 | 9 | 13 | 8 | 12 | 17 | 18 |
| 48 | 13 | 14 | 16 | 17 | 15 | 17 | 13 | 15 | 12 | 14 | 14 | 16 | 13 | 14 | 15 | 15 | 10 | 11 | 12 | 12 | 10 | 10 | 13 | 13 | 12 | 12 | 7 | 11 | 12 | 13 | 13 | 15 | 13 | 14 | 11 | 12 | 12 | 12 | 12 | 14 | 17 | 17 |
| 49 | 14 | 15 | 18 | 18 | 16 | 17 | 12 | 16 | 14 | 14 | 13 | 14 | 13 | 13 | 14 | 15 | 11 | 12 | 8 | 10 | 9 | 10 | 14.2 | 15.2 | 11 | 14 | 11 | 12 | 11 | 11 | 14 | 16 | 11 | 13 | 13 | 13 | 12 | 12 | 12 | 13 | 17 | 20 |
| 50 | 14 | 16 | 16 | 16 | 11 | 17 | 13 | 13 | 12 | 15 | 13 | 15 | 14 | 14 | 13 | 15 | 11 | 12 | 8 | 8 | 9 | 9 | 12 | 13 | 9 | 12 | 7 | 12 | 11 | 14 | 14 | 14 | 10 | 14 | 11 | 12 | 12 | 12 | 11 | 12 | 18 | 20 |
| 51 | 14 | 14 | 17 | 19 | 11 | 16 | 14 | 14 | 15 | 15 | 13 | 16 | 12 | 14 | 13 | 15 | 12 | 14 | 10 | 10 | 9 | 10 | 13 | 13.2 | 11 | 11 | 12 | 12 | 11 | 13 | 13 | 14 | 13 | 14 | 12 | 12 | 11 | 11 | 13 | 14 | 14 | 18 |
| 52 | 14 | 15 | 12 | 16 | 11 | 16 | 14 | 14 | 10 | 13 | 15 | 15 | 13 | 13 | 14 | 15 | 11 | 11 | 10 | 10 | 8 | 10 | 14 | 14.2 | 12 | 13 | 7 | 7 | 14 | 14 | 13 | 13 | 13 | 13 | 11 | 13 | 10 | 12 | 13 | 13 | 14 | 18 |
| 53 | 14 | 14 | 12 | 12 | 11 | 17 | 15 | 15 | 14 | 14 | 15 | 16 | 13 | 13 | 13 | 15 | 10 | 14 | 8 | 12 | 9 | 10 | 13 | 14 | 12 | 13 | 7 | 12 | 11 | 13 | 13 | 14 | 10 | 13 | 11 | 13 | 11 | 12 | 10 | 13 | 14 | 14 |
| 54 | 15 | 16 | 12 | 19 | 15 | 15 | 12 | 15 | 14 | 14 | 15 | 15 | 13 | 14 | 13 | 14 | 10 | 11 | 12 | 12 | 8 | 9 | 14 | 14 | 11 | 12 | 7 | 11 | 13 | 14 | 12 | 14 | 11 | 13 | 12 | 13 | 7 | 12 | 11 | 15 | 14 | 17 |
| 55 | 14 | 15 | 12 | 12 | 17 | 18 | 13 | 14 | 14 | 15 | 14 | 15 | 14 | 14 | 14 | 15 | 10 | 12 | 10 | 10 | 11 | 11 | 14 | 15.2 | 9 | 12 | 7 | 12 | 13 | 14 | 15 | 15 | 13 | 14 | 10 | 12 | 11 | 12 | 12 | 13 | 14 | 20 |
| 56 | 14 | 15 | 12 | 18 | 11 | 15 | 14 | 15 | 13 | 14 | 15 | 16 | 13 | 14 | 13 | 15 | 10 | 10 | 8 | 12 | 10 | 10 | 13 | 14.2 | 10 | 11 | 7 | 7 | 13 | 15 | 13 | 13 | 14 | 14 | 13 | 13 | 11 | 12 | 12 | 13 | 17 | 18 |
| 57 | 15 | 15 | 14 | 17 | 17 | 17 | 13 | 13 | 14 | 14 | 13 | 17 | 12 | 13 | 15 | 15 | 11 | 14 | 8 | 10 | 8 | 10 | 15 | 15 | 11 | 12 | 7 | 12 | 11 | 14 | 14 | 14 | 14 | 14 | 10 | 10 | 12 | 12 | 13 | 13 | 14 | 14 |
| 58 | 14 | 15 | 17 | 17 | 11 | 16 | 14 | 17 | 15 | 16 | 14 | 15 | 13 | 14 | 14 | 15 | 11 | 11 | 10 | 12 | 9 | 10 | 14 | 15 | 10 | 12 | 7 | 12 | 11 | 13 | 12 | 13 | 13 | 13 | 12 | 12 | 11 | 12 | 12 | 13 | 14 | 18 |
| 59 | 14 | 14 | 14 | 17 | 11 | 18 | 14 | 16 | 14 | 15 | 13 | 14 | 13 | 13 | 15 | 16 | 11 | 12 | 12 | 12 | 10 | 11 | 13 | 14 | 12 | 13 | 7 | 7 | 11 | 12 | 13 | 14 | 11 | 13 | 13 | 14 | 10 | 12 | 12 | 13 | 14 | 17 |
| 60 | 15 | 15 | 12 | 17 | 15 | 16 | 13 | 14 | 15 | 15 | 13 | 14 | 13 | 14 | 15 | 15 | 11 | 11 | 12 | 13 | 8 | 10 | 14 | 14.2 | 12 | 14 | 11 | 11 | 11 | 11 | 14 | 14 | 11 | 13 | 13 | 13 | 12 | 13 | 12 | 13 | 17 | 18 |
| 61 | 14 | 16 | 17 | 17 | 15 | 17 | 13 | 17 | 14 | 15 | 13 | 15 | 13 | 14 | 14 | 15 | 10 | 11 | 10 | 12 | 8 | 8 | 13 | 14 | 11 | 12 | 12 | 12 | 11 | 13 | 14 | 16 | 14 | 14 | 11 | 13 | 11 | 11 | 13 | 14 | 14 | 14 |
| 62 | 16 | 17 | 12 | 18 | 15 | 15 | 13 | 13 | 14 | 14 | 13 | 14 | 13 | 13 | 14 | 15 | 11 | 11 | 10 | 13 | 9 | 11 | 15 | 15.2 | 10 | 13 | 7 | 11 | 13 | 14 | 13 | 15 | 14 | 14 | 11 | 12 | 12 | 13 | 8 | 11 | 14 | 14 |
| 63 | 14 | 15 | 12 | 17 | 15 | 16 | 12 | 13 | 14 | 15 | 13 | 14 | 12 | 13 | 15 | 15 | 10 | 10 | 10 | 14 | 8 | 8 | 13 | 13 | 13 | 13 | 7 | 12 | 12 | 13 | 11 | 14 | 10 | 14 | 11 | 12 | 9 | 11 | 8 | 14 | 14 | 17 |
| 64 | 15 | 15 | 16 | 17 | 15 | 16 | 15 | 16 | 14 | 14 | 15 | 15 | 12 | 13 | 13 | 17 | 10 | 14 | 8 | 12 | 8 | 9 | 15 | 16 | 12 | 12 | 7 | 13 | 11 | 11 | 13 | 15 | 13 | 14 | 13 | 13 | 12 | 12 | 11 | 14 | 17 | 18 |
| 65 | 14 | 14 | 16 | 17 | 15 | 17 | 14 | 15 | 14 | 14 | 15 | 15 | 13 | 13 | 13 | 16 | 10 | 10 | 10 | 13 | 9 | 11 | 11 | 15.2 | 12 | 13 | 7 | 11 | 11 | 14 | 14 | 15 | 10 | 14 | 12 | 13 | 11 | 12 | 12 | 12 | 17 | 18 |
| 66 | 14 | 15 | 12 | 18 | 15 | 16 | 13 | 15 | 15 | 15 | 14 | 16 | 12 | 14 | 13 | 14 | 9.1 | 12 | 8 | 12 | 10 | 11 | 15 | 15 | 12 | 13 | 7 | 7 | 14 | 14 | 12 | 14 | 13 | 14 | 12 | 13 | 11 | 13 | 12 | 13 | 14 | 14 |
| 67 | 14 | 15 | 12 | 12 | 11 | 16 | 13 | 13 | 14 | 15 | 13 | 14 | 13 | 14 | 15 | 16 | 10 | 12 | 11 | 12 | 10 | 10 | 14.2 | 15.2 | 10 | 12 | 7 | 13 | 12 | 15 | 13 | 14 | 10 | 13 | 13 | 13 | 10 | 14 | 8 | 13 | 14 | 17 |
| 68 | 15 | 16 | 13 | 14 | 11 | 14 | 13 | 13 | 15 | 15 | 12 | 12 | 13 | 13 | 15 | 15 | 13 | 14 | 8 | 13 | 8 | 11 | 14 | 14.2 | 10 | 13 | 7 | 7 | 11 | 14 | 14 | 16 | 10 | 14 | 11 | 12 | 11 | 11 | 11 | 12 | 14 | 18 |
| 69 | 14 | 14 | 12 | 16 | 15 | 17 | 12 | 13 | 14 | 15 | 13 | 14 | 13 | 14 | 14 | 16 | 11 | 14 | 8 | 10 | 8 | 8 | 13.2 | 15 | 11 | 11 | 7 | 7 | 12 | 13 | 12 | 15 | 10 | 14 | 10 | 12 | 12 | 12 | 12 | 12 | 17 | 17 |
| 70 | 15 | 16 | 17 | 17 | 15 | 15 | 13 | 14 | 13 | 14 | 15 | 16 | 12 | 13 | 15 | 16 | 10 | 14 | 10 | 12 | 8 | 9 | 13 | 14 | 11 | 13 | 7 | 13 | 11 | 14 | 10 | 13 | 13 | 14 | 13 | 13 | 11 | 12 | 11 | 12 | 14 | 18 |
| 71 | 14 | 14 | 14 | 17 | 11 | 16 | 13 | 13 | 12 | 14 | 13 | 14 | 12 | 12 | 14 | 15 | 10 | 11 | 8 | 10 | 8 | 11 | 12 | 14.2 | 12 | 14 | 12 | 12 | 11 | 14 | 14 | 14 | 13 | 13 | 13 | 14 | 11 | 13 | 12 | 12 | 14 | 17 |
| 72 | 14 | 14 | 16 | 17 | 11 | 16 | 14 | 15 | 14 | 15 | 14 | 15 | 13 | 13 | 13 | 15 | 11 | 11 | 8 | 10 | 9 | 9 | 13 | 16.2 | 12 | 14 | 7 | 7 | 11 | 14 | 15 | 15 | 13 | 14 | 11 | 12 | 13 | 13 | 13 | 13 | 14 | 18 |
| 73 | 14 | 15 | 12 | 12 | 11 | 11 | 13 | 14 | 14 | 16 | 13 | 15 | 12 | 13 | 15 | 15 | 10 | 11 | 8 | 10 | 9 | 11 | 14 | 14 | 13 | 14 | 7 | 7 | 11 | 11 | 12 | 15 | 14 | 14 | 12 | 13 | 9 | 11 | 12 | 14 | 17 | 18 |
| 74 | 15 | 15 | 12 | 17 | 11 | 11 | 13 | 14 | 14 | 15 | 15 | 15 | 12 | 14 | 13 | 16 | 11 | 15 | 10 | 12 | 9 | 9 | 14 | 15.2 | 13 | 14 | 7 | 7 | 11 | 13 | 13 | 15 | 11 | 13 | 12 | 13 | 11 | 12 | 12 | 13 | 14 | 14 |
| 75 | 15 | 16 | 12 | 17 | 11 | 16 | 13 | 15 | 15 | 15 | 12 | 13 | 13 | 13 | 14 | 15 | 11 | 11 | 12 | 12 | 8 | 10 | 13 | 16 | 11 | 13 | 11 | 12 | 14 | 14 | 12 | 14 | 10 | 13 | 11 | 14 | 11 | 11 | 12 | 13 | 18 | 20 |
| 76 | 15 | 17 | 12 | 18 | 11 | 16 | 15 | 15 | 14 | 14 | 14 | 14 | 13 | 14 | 15 | 16 | 11 | 11 | 8 | 10 | 9 | 11 | 13 | 16 | 12 | 12 | 7 | 12 | 11 | 13 | 15 | 15 | 12 | 14 | 11 | 13 | 11 | 13 | 14 | 14 | 14 | 14 |
| 77 | 14 | 17 | 14 | 18 | 17 | 18 | 12 | 13 | 14 | 14 | 13 | 14 | 13 | 13 | 13 | 14 | 10 | 12 | 7 | 12 | 9 | 10 | 13 | 13 | 11 | 12 | 11 | 11 | 14 | 14 | 13 | 14 | 13 | 14 | 12 | 14 | 11 | 13 | 12 | 13 | 14 | 17 |
| 78 | 14 | 16 | 12 | 12 | 15 | 16 | 13 | 13 | 14 | 15 | 14 | 15 | 13 | 13 | 14 | 14 | 11.3 | 14 | 10 | 13 | 8 | 9 | 15.2 | 16 | 10 | 12 | 7 | 7 | 11 | 13 | 13 | 14 | 14 | 15 | 12 | 13 | 12 | 13 | 12 | 12 | 17 | 17 |
| 79 | 14 | 18 | 12 | 12 | 17 | 17 | 15 | 15 | 14 | 14 | 13 | 15 | 13 | 13 | 15 | 15 | 10 | 12 | 12 | 13 | 8 | 9 | 12 | 13 | 12 | 12 | 11 | 12 | 11 | 14 | 12 | 14 | 11 | 14 | 12 | 13 | 12 | 13 | 12 | 12 | 14 | 18 |
| 80 | 14 | 15 | 12 | 17 | 15 | 19 | 14 | 15 | 15 | 15 | 14 | 16 | 13 | 13 | 14 | 15 | 11 | 12 | 10 | 12 | 8 | 9 | 12 | 14.2 | 13 | 13 | 7 | 7 | 11 | 14 | 12 | 15 | 10 | 13 | 11 | 12 | 11 | 12 | 12 | 12 | 14 | 18 |
| 81 | 15 | 16 | 17 | 18 | 15 | 16 | 13 | 14 | 13 | 15 | 14 | 15 | 12 | 13 | 13 | 13 | 12 | 14 | 10 | 13 | 8 | 10 | 14 | 15.2 | 10 | 12 | 7 | 7 | 11 | 14 | 12 | 16 | 13 | 15 | 12 | 13 | 12 | 13 | 12 | 14 | 14 | 17 |
| 82 | 14 | 16 | 12 | 16 | 15 | 16 | 12 | 13 | 14 | 14 | 13 | 14 | 10 | 13 | 16 | 17 | 11 | 12 | 8 | 12 | 9 | 10 | 13 | 14 | 11 | 13 | 11 | 12 | 13 | 14 | 13 | 14 | 11 | 13 | 12 | 13 | 11 | 12 | 13 | 13 | 18 | 18 |
| Sample Name  Supplementary Table 1 (*Continued*)   |  |  |  |  |  |  |  |  |  |  |  |  |  |  |  |  |  |  |  |  |  |  |  |  |  |  |  |  |  |  |  |  |  |  |  |  |  |  |  |  |  |  |  | | --- | --- | --- | --- | --- | --- | --- | --- | --- | --- | --- | --- | --- | --- | --- | --- | --- | --- | --- | --- | --- | --- | --- | --- | --- | --- | --- | --- | --- | --- | --- | --- | --- | --- | --- | --- | --- | --- | --- | --- | --- | --- | --- | | D6S474 | D6S474 | D12ATA63 | D12ATA63 | D22S1045 | D22S1045 | D10S1248 | D10S1248 | D1S1677 | D1S1677 | D11S4463 | D11S4463 | D1S1627 | D1S1627 | D3S4529 | D3S4529 | D2S441 | D2S441 | D6S1017 | D6S1017 | D4S2408 | D4S2408 | D19S433 | D19S433 | D17S1301 | D17S1301 | D1GATA113 | D1GATA113 | D18S853 | D18S853 | D20S482 | D20S482 | D14S1434 | D14S1434 | D9S1122 | D9S1122 | D2S1776 | D2S1776 | D10S1435 | D10S1435 | D5S2500 | D5S2500 |
| 83 | 14 | 15 | 16 | 17 | 15 | 16 | 13 | 15 | 14 | 14 | 15 | 15 | 10 | 13 | 13 | 16 | 12 | 12 | 8 | 12 | 10 | 11 | 14 | 15 | 12 | 13 | 7 | 7 | 11 | 13 | 13 | 16 | 10 | 10 | 12 | 14 | 11 | 12 | 12 | 13 | 17 | 20 |
| 84 | 14 | 15 | 12 | 12 | 11 | 15 | 13 | 14 | 13 | 14 | 14 | 15 | 13 | 14 | 15 | 15 | 10 | 12 | 13 | 13 | 8 | 9 | 13 | 13.2 | 10 | 12 | 7 | 7 | 14 | 15 | 13 | 14 | 11 | 14 | 13 | 13 | 12 | 13 | 10 | 12 | 14 | 18 |
| 85 | 16 | 17 | 16 | 17 | 15 | 15 | 13 | 13 | 14 | 15 | 13 | 14 | 13 | 14 | 15 | 15 | 11 | 14 | 10 | 12 | 9 | 11 | 13 | 14 | 12 | 13 | 7 | 12 | 11 | 14 | 12 | 13 | 13 | 14 | 10 | 11 | 12 | 12 | 11 | 12 | 14 | 18 |
| 86 | 15 | 17 | 14 | 16 | 16 | 18 | 13 | 15 | 14 | 14 | 14 | 14 | 13 | 13 | 15 | 15 | 11 | 12 | 10 | 12 | 8 | 10 | 14 | 15 | 12 | 12 | 7 | 7 | 11 | 13 | 15 | 16 | 11 | 14 | 12 | 13 | 12 | 13 | 12 | 13 | 14 | 18 |
| 87 | 14 | 14 | 12 | 18 | 11 | 15 | 14 | 14 | 14 | 14 | 13 | 13 | 12 | 13 | 15 | 15 | 11 | 11 | 8 | 10 | 8 | 9 | 13.2 | 15.2 | 12 | 12 | 7 | 7 | 11 | 11 | 15 | 15 | 11 | 14 | 13 | 14 | 12 | 13 | 12 | 13 | 14 | 17 |
| 88 | 14 | 15 | 17 | 18 | 16 | 16 | 13 | 13 | 14 | 15 | 15 | 16 | 13 | 13 | 13 | 15 | 11 | 11 | 8 | 12 | 9 | 10 | 14 | 15.2 | 12 | 12 | 7 | 12 | 11 | 14 | 13 | 13 | 11 | 14 | 12 | 12 | 12 | 12 | 13 | 14 | 17 | 17 |
| 89 | 15 | 16 | 16 | 18 | 11 | 17 | 14 | 15 | 15 | 15 | 15 | 15 | 13 | 14 | 15 | 15 | 12 | 12 | 8 | 12 | 9 | 9 | 13 | 15.2 | 11 | 13 | 11 | 12 | 11 | 11 | 10 | 13 | 13 | 14 | 12 | 12 | 11 | 12 | 10 | 12 | 17 | 18 |
| 90 | 14 | 15 | 17 | 18 | 13 | 16 | 14 | 15 | 14 | 15 | 16 | 16 | 10 | 13 | 14 | 16 | 10 | 10 | 8 | 10 | 9 | 10 | 15.2 | 15.2 | 12 | 12 | 7 | 12 | 13 | 13 | 14 | 14 | 13 | 13 | 12 | 13 | 11 | 12 | 12 | 13 | 14 | 14 |
| 91 | 14 | 16 | 17 | 17 | 11 | 11 | 13 | 14 | 14 | 15 | 13 | 14 | 13 | 14 | 13 | 13 | 11 | 12 | 8 | 8 | 8 | 11 | 13 | 14 | 12 | 12 | 7 | 7 | 14 | 14 | 15 | 15 | 10 | 13 | 13 | 13 | 12 | 12 | 12 | 13 | 17 | 18 |
| 92 | 14 | 15 | 12 | 17 | 11 | 16 | 14 | 15 | 15 | 16 | 13 | 15 | 11 | 13 | 15 | 16 | 12 | 12 | 10 | 10 | 9 | 10 | 12 | 14.2 | 11 | 11 | 7 | 7 | 10 | 11 | 13 | 14 | 13 | 13 | 13 | 13 | 11 | 12 | 12 | 14 | 14 | 18 |
| 93 | 15 | 17 | 12 | 17 | 11 | 15 | 14 | 15 | 13 | 15 | 14 | 15 | 13 | 14 | 14 | 15 | 10 | 11 | 10 | 12 | 10 | 10 | 13 | 13 | 13 | 13 | 7 | 8 | 11 | 14 | 10 | 16 | 13 | 13 | 12 | 12 | 11 | 12 | 12 | 14 | 14 | 14 |
| 94 | 15 | 15 | 12 | 20 | 16 | 17 | 13 | 14 | 14 | 14 | 14 | 15 | 13 | 14 | 15 | 16 | 11 | 12 | 8 | 12 | 8 | 9 | 14 | 16 | 12 | 12 | 11 | 12 | 11 | 13 | 14 | 15 | 11 | 14 | 11 | 12 | 11 | 12 | 11 | 12 | 18 | 18 |
| 95 | 15 | 15 | 12 | 16 | 11 | 15 | 13 | 13 | 14 | 15 | 12 | 14 | 13 | 14 | 14 | 15 | 11 | 14 | 10 | 13 | 10 | 11 | 14 | 17.2 | 12 | 12 | 12 | 12 | 11 | 11 | 13 | 14 | 14 | 14 | 12 | 12 | 10 | 11 | 12 | 12 | 17 | 17 |
| 96 | 14 | 15 | 17 | 17 | 11 | 17 | 14 | 15 | 10 | 15 | 14 | 15 | 14 | 14 | 15 | 15 | 10 | 12 | 10 | 12 | 8 | 8 | 13.2 | 14 | 12 | 13 | 12 | 13 | 11 | 11 | 14 | 14 | 11 | 14 | 12 | 13 | 10 | 11 | 8 | 11 | 14 | 18 |
| 97 | 15 | 18 | 18 | 18 | 16 | 17 | 13 | 15 | 14 | 15 | 15 | 17 | 14 | 14 | 13 | 16 | 10 | 11 | 10 | 12 | 8 | 9 | 13 | 13 | 12 | 14 | 7 | 7 | 11 | 14 | 13 | 14 | 13 | 13 | 13 | 13 | 12 | 12 | 13 | 14 | 17 | 18 |
| 98 | 17 | 17 | 13 | 17 | 15 | 16 | 12 | 15 | 14 | 15 | 14 | 14 | 13 | 14 | 15 | 15 | 10.1 | 11 | 8 | 12 | 8 | 10 | 13 | 13 | 13 | 14 | 7 | 7 | 14 | 15 | 14 | 15 | 14 | 14 | 11 | 12 | 11 | 13 | 12 | 13 | 14 | 17 |
| 99 | 15 | 15 | 12 | 14 | 16 | 17 | 13 | 14 | 14 | 14 | 12 | 14 | 13 | 13 | 15 | 15 | 10 | 12 | 10 | 12 | 8 | 9 | 12 | 13 | 12 | 12 | 7 | 7 | 14 | 14 | 14 | 14 | 11 | 14 | 12 | 12 | 13 | 13 | 12 | 12 | 14 | 17 |
| 100 | 15 | 17 | 12 | 16 | 15 | 16 | 13 | 15 | 13 | 15 | 14 | 14 | 13 | 14 | 14 | 16 | 11 | 11 | 8 | 10 | 8 | 8 | 13 | 13.2 | 12 | 15 | 11 | 12 | 12 | 14 | 10 | 15 | 13 | 14 | 11 | 12 | 11 | 12 | 12 | 13 | 14 | 18 |
| 101 | 16 | 17 | 16 | 18 | 16 | 16 | 12 | 14 | 14 | 14 | 13 | 14 | 13 | 13 | 13 | 14 | 10 | 13 | 10 | 10 | 11 | 12 | 14 | 14 | 11 | 14 | 7 | 7 | 11 | 12 | 13 | 14 | 11 | 11 | 10 | 13 | 11 | 12 | 11 | 13 | 14 | 17 |
| 102 | 15 | 15 | 12 | 17 | 11 | 15 | 15 | 16 | 14 | 14 | 14 | 14 | 13 | 14 | 13 | 16 | 10.1 | 12 | 8 | 12 | 9 | 10 | 13 | 14.2 | 12 | 13 | 11 | 12 | 11 | 11 | 12 | 14 | 13 | 14 | 11 | 13 | 12 | 13 | 9 | 13 | 17 | 20 |
| 103 | 15 | 15 | 16 | 17 | 11 | 15 | 13 | 14 | 15 | 15 | 14 | 14 | 13 | 13 | 13 | 15 | 11.3 | 14 | 8 | 8 | 8 | 10 | 13 | 15 | 12 | 12 | 7 | 7 | 11 | 14 | 14 | 14 | 14 | 14 | 11 | 12 | 11 | 12 | 11 | 12 | 14 | 23 |
| 104 | 15 | 16 | 12 | 17 | 11 | 17 | 12 | 13 | 15 | 15 | 12 | 16 | 12 | 14 | 13 | 16 | 11 | 11 | 8 | 10 | 10 | 10 | 13 | 15 | 11 | 12 | 7 | 8 | 11 | 14 | 12 | 14 | 11 | 13 | 11 | 12 | 12 | 12 | 12 | 13 | 17 | 18 |
| 105 | 15 | 15 | 12 | 17 | 16 | 16 | 15 | 16 | 15 | 15 | 14 | 14 | 13 | 14 | 15 | 16 | 10 | 12 | 8 | 12 | 8 | 9 | 14 | 14 | 11 | 13 | 7 | 12 | 11 | 13 | 14 | 14 | 10 | 14 | 13 | 14 | 9 | 10 | 11 | 13 | 14 | 18 |
| 106 | 14 | 15 | 14 | 17 | 11 | 16 | 13 | 16 | 13 | 14 | 13 | 14 | 13 | 13 | 15 | 15 | 12 | 14 | 10 | 13 | 10 | 11 | 13 | 14 | 10 | 11 | 12 | 12 | 12 | 13 | 14 | 14 | 13 | 14 | 12 | 12 | 9 | 12 | 12 | 12 | 14 | 14 |
| 107 | 14 | 15 | 12 | 18 | 11 | 16 | 12 | 13 | 14 | 15 | 13 | 13 | 11 | 13 | 13 | 14 | 10 | 10 | 10 | 12 | 10 | 10 | 13 | 14 | 11 | 12 | 11 | 13 | 11 | 11 | 13 | 15 | 10 | 14 | 12 | 13 | 11 | 13 | 11 | 15 | 17 | 18 |
| 108 | 15 | 16 | 12 | 17 | 16 | 17 | 14 | 15 | 14 | 15 | 14 | 18 | 13 | 13 | 14 | 15 | 10 | 11 | 10 | 10 | 9 | 10 | 14 | 14 | 10 | 15 | 11 | 11 | 11 | 14 | 10 | 13 | 13 | 14 | 10 | 11 | 9 | 10 | 11 | 13 | 14 | 17 |
| 109 | 15 | 18 | 17 | 18 | 15 | 16 | 13 | 16 | 14 | 15 | 16 | 17 | 14 | 14 | 16 | 16 | 11 | 14 | 10 | 10 | 8 | 9 | 13 | 13.2 | 12 | 13 | 10 | 12 | 11 | 14 | 13 | 14 | 11 | 14 | 12 | 12 | 12 | 12 | 13 | 15 | 17 | 17 |
| 110 | 15 | 16 | 12 | 17 | 15 | 17 | 15 | 15 | 13 | 14 | 15 | 15 | 13 | 14 | 15 | 15 | 10 | 14 | 11 | 13 | 8 | 8 | 13 | 13 | 12 | 12 | 7 | 12 | 11 | 13 | 14 | 14 | 14 | 14 | 12 | 12 | 12 | 12 | 11 | 12 | 17 | 23 |
| 111 | 14 | 14 | 12 | 18 | 11 | 17 | 13 | 13 | 14 | 15 | 13 | 14 | 13 | 14 | 13 | 15 | 11 | 11 | 10 | 10 | 10 | 10 | 15.2 | 15.2 | 12 | 13 | 12 | 13 | 13 | 13 | 13 | 16 | 13 | 14 | 10 | 12 | 10 | 12 | 12 | 13 | 14 | 17 |
| 112 | 14 | 15 | 12 | 12 | 15 | 17 | 14 | 15 | 15 | 15 | 13 | 14 | 14 | 14 | 14 | 15 | 10.1 | 10.1 | 10 | 10 | 10 | 10 | 13 | 16.2 | 12 | 12 | 7 | 7 | 11 | 14 | 12 | 15 | 13 | 14 | 11 | 14 | 11 | 12 | 11 | 13 | 14 | 17 |
| 113 | 14 | 15 | 12 | 16 | 11 | 17 | 14 | 15 | 14 | 15 | 15 | 15 | 12 | 13 | 14 | 15 | 11 | 11 | 10 | 13 | 10 | 10 | 12 | 14.2 | 11 | 12 | 7 | 7 | 13 | 14 | 13 | 14 | 13 | 15 | 12 | 13 | 9 | 13 | 12 | 13 | 14 | 14 |
| 114 | 15 | 16 | 12 | 12 | 15 | 15 | 13 | 13 | 14 | 14 | 14 | 14 | 13 | 13 | 15 | 15 | 10.1 | 11 | 10 | 12 | 8 | 8 | 13 | 13 | 12 | 13 | 7 | 11 | 11 | 13 | 11 | 14 | 13 | 13 | 12 | 13 | 11 | 13 | 11 | 12 | 17 | 18 |
| 115 | 14 | 15 | 12 | 16 | 11 | 17 | 13 | 15 | 12 | 15 | 14 | 15 | 13 | 14 | 13 | 16 | 12 | 12 | 10 | 10 | 8 | 8 | 13 | 15.2 | 11 | 13 | 11 | 12 | 11 | 14 | 14 | 14 | 13 | 14 | 13 | 14 | 11 | 12 | 10 | 12 | 14 | 17 |
| 116 | 14 | 15 | 16 | 17 | 11 | 16 | 12 | 13 | 15 | 15 | 14 | 14 | 13 | 14 | 16 | 17 | 11 | 11 | 8 | 8 | 10 | 11 | 14 | 15.2 | 11 | 12 | 7 | 12 | 13 | 14 | 13 | 15 | 11 | 12 | 11 | 13 | 9 | 14 | 11 | 13 | 14 | 17 |
| 117 | 15 | 15 | 12 | 17 | 15 | 15 | 14 | 17 | 13 | 14 | 14 | 14 | 13 | 14 | 14 | 15 | 10 | 11 | 12 | 13 | 8 | 10 | 13 | 15.2 | 12 | 12 | 7 | 11 | 13 | 14 | 10 | 14 | 12 | 14 | 11 | 13 | 10 | 13 | 12 | 13 | 14 | 18 |
| 118 | 14 | 14 | 12 | 16 | 15 | 16 | 13 | 15 | 14 | 15 | 14 | 16 | 13 | 14 | 13 | 16 | 12 | 14 | 11 | 12 | 10 | 10 | 13 | 13 | 12 | 13 | 7 | 13 | 11 | 13 | 14 | 16 | 13 | 14 | 12 | 13 | 13 | 13 | 11 | 12 | 18 | 18 |
| 119 | 15 | 16 | 16 | 17 | 15 | 16 | 14 | 15 | 14 | 15 | 14 | 15 | 13 | 14 | 13 | 15 | 11 | 12 | 12 | 12 | 9 | 9 | 14 | 15.2 | 11 | 12 | 7 | 12 | 12 | 15 | 13 | 16 | 10 | 14 | 10 | 12 | 11 | 12 | 13 | 14 | 14 | 14 |
| 120 | 15 | 17 | 12 | 12 | 11 | 16 | 14 | 15 | 14 | 15 | 15 | 15 | 13 | 13 | 15 | 15 | 11 | 12 | 10 | 12 | 11 | 11 | 13 | 15 | 11 | 13 | 7 | 11 | 12 | 13 | 13 | 15 | 10 | 13 | 11 | 13 | 11 | 11 | 10 | 11 | 17 | 18 |
| 121 | 15 | 16 | 12 | 12 | 11 | 17 | 13 | 15 | 14 | 14 | 12 | 14 | 13 | 14 | 15 | 15 | 10 | 12 | 10 | 10 | 10 | 11 | 14 | 15.2 | 11 | 12 | 11 | 12 | 11 | 15 | 14 | 14 | 13 | 13 | 12 | 12 | 9 | 11 | 13 | 14 | 14 | 18 |
| 122 | 14 | 17 | 12 | 18 | 11 | 16 | 14 | 15 | 14 | 14 | 15 | 15 | 12 | 13 | 15 | 15 | 11 | 11 | 10 | 12 | 8 | 12 | 13 | 14 | 10 | 12 | 7 | 11 | 13 | 15 | 14 | 16 | 10 | 12 | 11 | 14 | 12 | 13 | 12 | 13 | 14 | 17 |
| 123 | 14 | 16 | 16 | 17 | 15 | 16 | 13 | 15 | 14 | 15 | 13 | 14 | 13 | 14 | 15 | 16 | 10 | 10 | 10 | 12 | 8 | 10 | 15.2 | 15.2 | 12 | 14 | 7 | 7 | 13 | 14 | 14 | 14 | 13 | 13 | 12 | 12 | 9 | 10 | 10 | 10 | 14 | 17 |
| Sample Name  Supplementary Table 1 (*Continued*)   |  |  |  |  |  |  |  |  |  |  |  |  |  |  |  |  |  |  |  |  |  |  |  |  |  |  |  |  |  |  |  |  |  |  |  |  |  |  |  |  |  |  |  | | --- | --- | --- | --- | --- | --- | --- | --- | --- | --- | --- | --- | --- | --- | --- | --- | --- | --- | --- | --- | --- | --- | --- | --- | --- | --- | --- | --- | --- | --- | --- | --- | --- | --- | --- | --- | --- | --- | --- | --- | --- | --- | --- | | D6S474 | D6S474 | D12ATA63 | D12ATA63 | D22S1045 | D22S1045 | D10S1248 | D10S1248 | D1S1677 | D1S1677 | D11S4463 | D11S4463 | D1S1627 | D1S1627 | D3S4529 | D3S4529 | D2S441 | D2S441 | D6S1017 | D6S1017 | D4S2408 | D4S2408 | D19S433 | D19S433 | D17S1301 | D17S1301 | D1GATA113 | D1GATA113 | D18S853 | D18S853 | D20S482 | D20S482 | D14S1434 | D14S1434 | D9S1122 | D9S1122 | D2S1776 | D2S1776 | D10S1435 | D10S1435 | D5S2500 | D5S2500 |
| 124 | 14 | 15 | 12 | 12 | 15 | 15 | 13 | 13 | 12 | 14 | 14 | 15 | 13 | 13 | 14 | 14 | 13 | 14 | 10 | 12 | 9 | 11 | 14 | 16.2 | 11 | 14 | 7 | 12 | 11 | 11 | 14 | 14 | 10 | 13 | 11 | 13 | 9 | 12 | 11 | 12 | 14 | 14 |
| 125 | 15 | 15 | 17 | 17 | 15 | 17 | 13 | 15 | 14 | 16 | 15 | 16 | 11 | 13 | 13 | 16 | 11 | 12 | 12 | 12 | 10 | 10 | 14 | 14 | 12 | 14 | 7 | 12 | 11 | 11 | 10 | 16 | 11 | 14 | 10 | 11 | 9 | 11 | 12 | 14 | 17 | 18 |
| 126 | 14 | 14 | 14 | 18 | 15 | 15 | 13 | 16 | 14 | 15 | 13 | 16 | 12 | 13 | 13 | 15 | 10 | 11 | 8 | 8 | 9 | 11 | 14 | 15 | 12 | 12 | 11 | 11 | 11 | 15 | 14 | 16 | 11 | 13 | 11 | 14 | 10 | 10 | 12 | 12 | 17 | 17 |
| 127 | 15 | 18 | 16 | 17 | 11 | 15 | 14 | 15 | 14 | 15 | 14 | 16 | 14 | 14 | 15 | 15 | 11 | 11 | 8 | 13 | 9 | 11 | 14 | 14 | 10 | 12 | 7 | 12 | 13 | 14 | 14 | 14 | 11 | 13 | 11 | 12 | 11 | 11 | 12 | 14 | 14 | 20 |
| 128 | 14 | 15 | 17 | 18 | 15 | 16 | 13 | 15 | 14 | 14 | 15 | 15 | 13 | 13 | 13 | 13 | 10 | 12 | 8 | 10 | 8 | 10 | 13 | 14 | 11 | 14 | 12 | 12 | 10 | 13 | 14 | 15 | 11 | 16 | 11 | 12 | 11 | 12 | 14 | 15 | 14 | 18 |
| 129 | 14 | 17 | 16 | 17 | 16 | 16 | 13 | 13 | 13 | 14 | 13 | 14 | 13 | 13 | 13 | 15 | 11 | 11 | 10 | 12 | 8 | 11 | 13 | 15.2 | 12 | 14 | 7 | 12 | 13 | 13 | 13 | 14 | 13 | 14 | 11 | 13 | 9 | 12 | 12 | 12 | 14 | 17 |
| 130 | 14 | 15 | 12 | 17 | 15 | 16 | 13 | 14 | 14 | 14 | 15 | 16 | 13 | 14 | 15 | 16 | 11 | 11 | 10 | 13 | 8 | 9 | 13 | 15 | 11 | 11 | 7 | 12 | 11 | 13 | 13 | 13 | 11 | 14 | 12 | 14 | 12 | 13 | 12 | 13 | 18 | 23 |
| 131 | 15 | 17 | 12 | 12 | 11 | 16 | 13 | 13 | 12 | 13 | 13 | 14 | 13 | 14 | 15 | 16 | 11 | 11 | 10 | 11 | 9 | 12 | 14 | 14 | 12 | 12 | 7 | 7 | 14 | 14 | 14 | 16 | 11 | 13 | 10 | 12 | 11 | 12 | 12 | 14 | 17 | 18 |
| 132 | 14 | 14 | 17 | 17 | 11 | 17 | 13 | 14 | 14 | 14 | 14 | 15 | 13 | 13 | 15 | 16 | 10 | 11 | 8 | 10 | 10 | 10 | 14 | 14.2 | 11 | 12 | 7 | 12 | 12 | 13 | 14 | 15 | 10 | 14 | 12 | 14 | 12 | 13 | 11 | 12 | 14 | 18 |
| 133 | 14 | 17 | 12 | 17 | 15 | 15 | 13 | 13 | 14 | 15 | 15 | 17 | 13 | 13 | 13 | 15 | 11 | 11 | 10 | 13 | 9 | 10 | 14.2 | 15 | 12 | 12 | 7 | 12 | 11 | 14 | 13 | 14 | 11 | 13 | 11 | 13 | 10 | 12 | 13 | 14 | 14 | 14 |
| 134 | 14 | 14 | 17 | 18 | 12 | 16 | 15 | 16 | 14 | 14 | 15 | 16 | 13 | 14 | 15 | 15 | 10 | 11 | 8 | 8 | 8 | 10 | 13 | 13.2 | 10 | 14 | 7 | 11 | 11 | 14 | 13 | 15 | 13 | 14 | 11 | 13 | 12 | 12 | 14 | 14 | 18 | 20 |
| 135 | 15 | 15 | 12 | 12 | 15 | 16 | 15 | 15 | 15 | 15 | 12 | 14 | 13 | 13 | 13 | 15 | 10 | 10 | 8 | 10 | 11 | 11 | 13 | 16.2 | 11 | 13 | 12 | 12 | 13 | 13 | 11 | 15 | 10 | 13 | 11 | 13 | 12 | 12 | 12 | 13 | 14 | 17 |
| 136 | 14 | 14 | 17 | 17 | 15 | 15 | 14 | 16 | 14 | 14 | 15 | 15 | 13 | 13 | 13 | 17 | 10 | 11 | 10 | 12 | 9 | 10 | 12 | 14.2 | 9 | 12 | 7 | 12 | 11 | 13 | 12 | 16 | 11 | 14 | 10 | 10 | 11 | 11 | 11 | 11 | 14 | 17 |
| 137 | 14 | 15 | 12 | 17 | 11 | 15 | 15 | 15 | 14 | 16 | 14 | 16 | 13 | 13 | 14 | 16 | 10 | 14 | 10 | 12 | 8 | 9 | 13 | 14 | 11 | 12 | 7 | 8 | 13 | 14 | 13 | 14 | 14 | 14 | 11 | 11 | 11 | 12 | 12 | 12 | 17 | 18 |
| 138 | 15 | 16 | 12 | 14 | 11 | 16 | 14 | 15 | 14 | 15 | 15 | 15 | 13 | 14 | 15 | 15 | 11 | 12 | 8 | 12 | 8 | 12 | 14 | 14 | 11 | 13 | 7 | 12 | 14 | 14 | 14 | 15 | 13 | 14 | 13 | 14 | 11 | 13 | 12 | 13 | 14 | 17 |
| 139 | 14 | 15 | 16 | 17 | 15 | 18 | 13 | 15 | 14 | 14 | 14 | 15 | 11 | 12 | 14 | 16 | 11 | 15 | 10 | 13 | 8 | 10 | 13 | 15.2 | 9 | 12 | 12 | 12 | 11 | 13 | 14 | 15 | 13 | 14 | 13 | 13 | 12 | 12 | 8 | 13 | 14 | 17 |
| 140 | 14 | 15 | 17 | 17 | 16 | 17 | 12 | 14 | 14 | 15 | 14 | 15 | 13 | 13 | 14 | 15 | 10 | 14 | 10 | 12 | 8 | 9 | 14.2 | 15 | 12 | 13 | 7 | 7 | 11 | 15 | 12 | 13 | 14 | 14 | 12 | 13 | 9 | 12 | 12 | 15 | 17 | 17 |
| 141 | 15 | 17 | 17 | 17 | 11 | 15 | 14 | 15 | 13 | 15 | 13 | 14 | 12 | 14 | 14 | 15 | 11 | 12 | 10 | 11 | 8 | 9 | 13 | 13 | 12 | 13 | 12 | 12 | 13 | 15 | 14 | 16 | 11 | 13 | 12 | 13 | 9 | 12 | 9 | 13 | 14 | 17 |
| 142 | 14 | 16 | 12 | 17 | 16 | 17 | 14 | 15 | 14 | 16 | 13 | 14 | 12 | 13 | 14 | 15 | 10 | 11 | 8 | 12 | 9 | 10 | 13.2 | 14 | 11 | 13 | 7 | 12 | 11 | 14 | 14 | 15 | 11 | 14 | 12 | 12 | 11 | 13 | 11 | 16 | 14 | 14 |
| 143 | 14 | 14 | 17 | 17 | 11 | 11 | 13 | 13 | 13 | 15 | 13 | 14 | 13 | 14 | 13 | 14 | 11 | 15 | 10 | 13 | 8 | 11 | 13 | 14 | 12 | 13 | 7 | 12 | 11 | 11 | 15 | 15 | 12 | 13 | 12 | 13 | 10 | 12 | 12 | 13 | 14 | 23 |
| 144 | 14 | 15 | 12 | 17 | 16 | 16 | 11 | 13 | 14 | 15 | 13 | 16 | 12 | 14 | 14 | 14 | 10 | 11 | 13 | 13 | 8 | 12 | 14 | 15 | 12 | 12 | 7 | 7 | 11 | 13 | 14 | 15 | 10 | 13 | 12 | 14 | 9 | 13 | 12 | 12 | 14 | 18 |
| 145 | 16 | 18 | 12 | 17 | 11 | 17 | 14 | 15 | 14 | 14 | 13 | 14 | 13 | 13 | 13 | 14 | 9.1 | 11 | 8 | 12 | 8 | 10 | 14 | 15 | 9 | 11 | 7 | 11 | 13 | 15 | 14 | 15 | 13 | 14 | 12 | 13 | 11 | 12 | 11 | 14 | 20 | 20 |
| 146 | 15 | 16 | 12 | 17 | 15 | 16 | 14 | 15 | 15 | 16 | 13 | 14 | 13 | 14 | 13 | 15 | 11 | 11.3 | 10 | 10 | 10 | 12 | 13 | 13 | 12 | 12 | 8 | 12 | 14 | 14 | 14 | 15 | 11 | 11 | 13 | 13 | 9 | 11 | 11 | 13 | 18 | 18 |
| 147 | 15 | 15 | 14 | 16 | 15 | 16 | 15 | 16 | 14 | 16 | 13 | 14 | 14 | 14 | 15 | 15 | 10 | 11 | 10 | 13 | 9 | 10 | 13 | 13 | 11 | 12 | 7 | 7 | 11 | 14 | 13 | 14 | 11 | 14 | 11 | 13 | 11 | 12 | 12 | 15 | 17 | 18 |
| 148 | 14 | 14 | 16 | 17 | 15 | 16 | 13 | 15 | 15 | 16 | 14 | 15 | 12 | 13 | 15 | 15 | 10 | 12 | 8 | 10 | 9 | 11 | 14 | 15.2 | 11 | 12 | 7 | 7 | 11 | 11 | 13 | 13 | 10 | 11 | 12 | 14 | 12 | 12 | 12 | 14 | 14 | 17 |
| 149 | 15 | 17 | 16 | 17 | 16 | 18 | 12 | 14 | 15 | 15 | 16 | 17 | 13 | 13 | 15 | 16 | 10 | 12 | 10 | 12 | 8 | 8 | 15.2 | 15.2 | 12 | 12 | 7 | 7 | 11 | 13 | 13 | 14 | 10 | 14 | 13 | 13 | 12 | 13 | 13 | 15 | 14 | 23 |
| 150 | 15 | 16 | 12 | 16 | 15 | 17 | 12 | 14 | 14 | 16 | 13 | 13 | 13 | 14 | 15 | 16 | 11 | 12 | 10 | 12 | 8 | 8 | 13 | 16.2 | 12 | 12 | 7 | 11 | 11 | 14 | 14 | 14 | 13 | 14 | 13 | 13 | 11 | 11 | 11 | 13 | 17 | 23 |
| 151 | 14 | 14 | 17 | 18 | 11 | 16 | 11 | 13 | 13 | 15 | 14 | 14 | 14 | 14 | 15 | 15 | 10 | 11 | 10 | 12 | 8 | 11 | 14 | 14.2 | 11 | 12 | 7 | 11 | 11 | 15 | 14 | 16 | 14 | 14 | 11 | 13 | 9 | 11 | 12 | 13 | 14 | 20 |
| 152 | 14 | 16 | 12 | 16 | 15 | 16 | 13 | 14 | 14 | 15 | 13 | 14 | 14 | 14 | 14 | 15 | 10 | 14 | 10 | 12 | 11 | 11 | 13.2 | 16 | 11 | 13 | 7 | 11 | 11 | 13 | 12 | 12 | 11 | 14 | 10 | 11 | 11 | 13 | 11 | 12 | 14 | 18 |
| 153 | 15 | 18 | 16 | 17 | 11 | 17 | 13 | 13 | 15 | 16 | 13 | 16 | 13 | 14 | 13 | 13 | 11.3 | 12 | 12 | 13 | 9 | 11 | 15 | 15.2 | 8 | 12 | 7 | 7 | 11 | 14 | 14 | 15 | 11 | 13 | 13 | 13 | 10 | 12 | 12 | 12 | 14 | 14 |
| 154 | 14 | 18 | 12 | 12 | 11 | 17 | 12 | 13 | 12 | 17 | 14 | 15 | 13 | 13 | 14 | 15 | 13 | 14 | 10 | 10 | 9 | 9 | 12 | 15.2 | 11 | 13 | 7 | 11 | 12 | 13 | 13 | 13 | 13 | 13 | 12 | 13 | 12 | 13 | 12 | 13 | 14 | 14 |
| 155 | 16 | 16 | 12 | 16 | 15 | 15 | 13 | 15 | 14 | 15 | 12 | 15 | 14 | 14 | 13 | 15 | 11 | 11 | 10 | 13 | 9 | 9 | 13 | 15.2 | 12 | 12 | 7 | 7 | 11 | 13 | 12 | 13 | 13 | 14 | 13 | 13 | 11 | 12 | 11 | 12 | 14 | 14 |
| 156 | 14 | 14 | 12 | 16 | 11 | 16 | 14 | 16 | 14 | 14 | 13 | 14 | 12 | 13 | 13 | 15 | 10 | 11.3 | 8 | 12 | 8 | 9 | 13 | 14 | 12 | 15 | 7 | 7 | 11 | 14 | 12 | 15 | 14 | 15 | 13 | 13 | 9 | 11 | 13 | 14 | 14 | 14 |
| 157 | 17 | 17 | 17 | 17 | 15 | 16 | 12 | 14 | 14 | 14 | 15 | 16 | 12 | 14 | 13 | 13 | 10 | 12 | 8 | 12 | 8 | 9 | 13 | 13.2 | 12 | 13 | 7 | 12 | 11 | 14 | 13 | 14 | 14 | 14 | 11 | 11 | 9 | 9 | 12 | 14 | 14 | 18 |
| 158 | 15 | 16 | 12 | 17 | 16 | 16 | 15 | 15 | 14 | 15 | 14 | 15 | 13 | 14 | 15 | 15 | 11 | 11 | 8 | 8 | 10 | 10 | 13 | 14 | 10 | 10 | 7 | 12 | 14 | 14 | 14 | 16 | 13 | 14 | 11 | 13 | 11 | 12 | 13 | 14 | 17 | 17 |
| 159 | 14 | 15 | 16 | 17 | 15 | 16 | 14 | 15 | 15 | 15 | 12 | 13 | 13 | 14 | 14 | 17 | 11 | 14 | 10 | 11 | 8 | 9 | 14.2 | 15 | 12 | 13 | 7 | 7 | 11 | 13 | 13 | 15 | 11 | 14 | 13 | 13 | 11 | 12 | 12 | 14 | 14 | 17 |
| 160 | 14 | 14 | 12 | 17 | 15 | 15 | 12 | 14 | 14 | 14 | 13 | 14 | 13 | 14 | 15 | 16 | 11.3 | 12 | 10 | 12 | 8 | 10 | 14.2 | 15.2 | 11 | 12 | 7 | 11 | 13 | 14 | 12 | 13 | 14 | 14 | 13 | 13 | 9 | 14 | 13 | 13 | 14 | 14 |
| 161 | 15 | 15 | 12 | 18 | 11 | 17 | 13 | 14 | 14 | 15 | 14 | 17 | 13 | 13 | 13 | 15 | 11 | 12 | 8 | 10 | 8 | 9 | 13 | 13 | 11 | 13 | 7 | 12 | 13 | 13 | 14 | 15 | 13 | 14 | 11 | 11 | 9 | 12 | 13 | 13 | 17 | 17 |
| 162 | 14 | 15 | 17 | 17 | 15 | 16 | 13 | 14 | 14 | 14 | 15 | 17 | 12 | 14 | 15 | 15 | 10 | 14 | 10 | 10 | 10 | 10 | 14 | 14.2 | 12 | 13 | 7 | 7 | 11 | 13 | 13 | 13 | 11 | 14 | 10 | 13 | 11 | 11 | 12 | 13 | 14 | 18 |
| 163 | 14 | 15 | 12 | 14 | 15 | 16 | 13 | 16 | 14 | 15 | 14 | 15 | 13 | 14 | 14 | 16 | 10 | 12 | 10 | 12 | 10 | 10 | 14 | 15 | 12 | 13 | 11 | 12 | 11 | 13 | 14 | 14 | 14 | 14 | 11 | 12 | 12 | 12 | 11 | 12 | 14 | 17 |
| 164 | 14 | 15 | 12 | 17 | 11 | 14 | 13 | 13 | 13 | 15 | 12 | 16 | 13 | 13 | 15 | 16 | 13 | 14 | 8 | 10 | 8 | 8 | 13 | 14 | 12 | 13 | 7 | 11 | 14 | 14 | 14 | 16 | 10 | 13 | 11 | 12 | 11 | 12 | 11 | 13 | 14 | 18 |
| Sample Name  Supplementary Table 1 (*Continued*)   |  |  |  |  |  |  |  |  |  |  |  |  |  |  |  |  |  |  |  |  |  |  |  |  |  |  |  |  |  |  |  |  |  |  |  |  |  |  |  |  |  |  |  | | --- | --- | --- | --- | --- | --- | --- | --- | --- | --- | --- | --- | --- | --- | --- | --- | --- | --- | --- | --- | --- | --- | --- | --- | --- | --- | --- | --- | --- | --- | --- | --- | --- | --- | --- | --- | --- | --- | --- | --- | --- | --- | --- | | D6S474 | D6S474 | D12ATA63 | D12ATA63 | D22S1045 | D22S1045 | D10S1248 | D10S1248 | D1S1677 | D1S1677 | D11S4463 | D11S4463 | D1S1627 | D1S1627 | D3S4529 | D3S4529 | D2S441 | D2S441 | D6S1017 | D6S1017 | D4S2408 | D4S2408 | D19S433 | D19S433 | D17S1301 | D17S1301 | D1GATA113 | D1GATA113 | D18S853 | D18S853 | D20S482 | D20S482 | D14S1434 | D14S1434 | D9S1122 | D9S1122 | D2S1776 | D2S1776 | D10S1435 | D10S1435 | D5S2500 | D5S2500 |
| 165 | 14 | 15 | 12 | 17 | 11 | 16 | 15 | 16 | 14 | 14 | 13 | 17 | 13 | 14 | 15 | 15 | 11 | 11 | 8 | 10 | 9 | 11 | 13 | 14.2 | 11 | 12 | 7 | 7 | 11 | 13 | 13 | 13 | 11 | 11 | 11 | 13 | 12 | 12 | 11 | 12 | 17 | 17 |
| 166 | 14 | 18 | 17 | 18 | 15 | 16 | 13 | 16 | 14 | 15 | 14 | 16 | 13 | 13 | 15 | 15 | 10 | 12 | 10 | 10 | 8 | 8 | 14 | 15.2 | 13 | 14 | 7 | 12 | 12 | 13 | 12 | 14 | 11 | 13 | 12 | 12 | 11 | 12 | 12 | 13 | 18 | 18 |
| 167 | 14 | 17 | 14 | 17 | 17 | 17 | 14 | 15 | 14 | 15 | 15 | 16 | 13 | 13 | 14 | 15 | 12 | 14 | 8 | 12 | 11 | 11 | 14 | 15 | 10 | 12 | 7 | 12 | 11 | 11 | 13 | 14 | 10 | 13 | 11 | 11 | 11 | 12 | 11 | 13 | 14 | 18 |
| 168 | 14 | 17 | 12 | 12 | 15 | 16 | 16 | 17 | 13 | 14 | 11 | 15 | 13 | 14 | 13 | 14 | 9.1 | 15 | 10 | 12 | 9 | 10 | 13 | 15.2 | 11 | 13 | 7 | 7 | 11 | 13 | 13 | 14 | 13 | 16 | 12 | 13 | 11 | 12 | 12 | 12 | 17 | 18 |
| 169 | 14 | 17 | 16 | 17 | 16 | 18 | 13 | 14 | 12 | 13 | 14 | 15 | 14 | 14 | 13 | 14 | 9.1 | 11 | 10 | 12 | 9 | 10 | 13 | 16.2 | 11 | 14 | 7 | 12 | 14 | 14 | 15 | 15 | 10 | 11 | 13 | 13 | 10 | 13 | 12 | 12 | 14 | 17 |
| 170 | 15 | 15 | 12 | 19 | 11 | 15 | 13 | 14 | 15 | 16 | 12 | 14 | 13 | 14 | 15 | 15 | 10 | 12 | 10 | 12 | 8 | 11 | 14.2 | 15 | 12 | 13 | 7 | 7 | 11 | 14 | 13 | 14 | 11 | 13 | 10 | 11 | 11 | 12 | 10 | 13 | 14 | 17 |
| 171 | 15 | 16 | 12 | 17 | 15 | 15 | 13 | 14 | 14 | 15 | 13 | 14 | 13 | 15 | 14 | 15 | 12 | 12 | 8 | 10 | 9 | 10 | 13 | 15.2 | 10 | 13 | 11 | 11 | 14 | 14 | 14 | 14 | 13 | 13 | 13 | 13 | 14 | 14 | 13 | 14 | 14 | 18 |
| 172 | 14 | 15 | 16 | 17 | 15 | 16 | 13 | 14 | 14 | 15 | 12 | 16 | 13 | 13 | 14 | 15 | 10 | 11 | 8 | 11 | 9 | 10 | 13 | 14 | 12 | 14 | 7 | 12 | 11 | 13 | 13 | 14 | 10 | 13 | 11 | 11 | 11 | 13 | 12 | 12 | 14 | 18 |
| 173 | 14 | 17 | 17 | 17 | 15 | 17 | 12 | 13 | 13 | 14 | 13 | 15 | 11 | 13 | 13 | 15 | 10 | 14 | 10 | 12 | 9 | 10 | 13 | 14 | 12 | 12 | 12 | 12 | 11 | 11 | 16 | 16 | 10 | 13 | 12 | 12 | 9 | 11 | 11 | 14 | 17 | 18 |
| 174 | 14 | 14 | 17 | 18 | 11 | 15 | 13 | 14 | 14 | 15 | 12 | 13 | 13 | 13 | 14 | 14 | 10 | 12 | 8 | 12 | 8 | 10 | 13 | 14 | 11 | 12 | 7 | 11 | 11 | 13 | 13 | 14 | 11 | 14 | 13 | 13 | 13 | 14 | 11 | 12 | 17 | 18 |
| 175 | 14 | 16 | 12 | 16 | 11 | 18 | 14 | 15 | 14 | 14 | 13 | 16 | 13 | 14 | 14 | 15 | 9.1 | 11 | 8 | 8 | 10 | 11 | 15 | 15.2 | 12 | 12 | 7 | 7 | 11 | 12 | 13 | 15 | 11 | 14 | 12 | 13 | 11 | 12 | 11 | 13 | 14 | 18 |
| 176 | 14 | 15 | 12 | 17 | 16 | 17 | 13 | 15 | 14 | 15 | 14 | 16 | 13 | 13 | 15 | 15 | 10 | 12 | 12 | 12 | 9 | 11 | 14 | 15 | 11 | 12 | 7 | 11 | 11 | 14 | 10 | 13 | 13 | 14 | 12 | 15 | 11 | 12 | 11 | 14 | 14 | 17 |
| 177 | 15 | 17 | 12 | 16 | 15 | 16 | 14 | 14 | 13 | 14 | 13 | 15 | 13 | 14 | 16 | 16 | 11 | 14 | 10 | 12 | 9 | 10 | 14.2 | 14.2 | 12 | 13 | 7 | 11 | 11 | 13 | 13 | 14 | 13 | 14 | 11 | 12 | 11 | 12 | 12 | 12 | 18 | 20 |
| 178 | 14 | 15 | 12 | 12 | 11 | 15 | 13 | 14 | 13 | 14 | 14 | 15 | 14 | 14 | 13 | 15 | 11 | 12 | 8 | 8 | 10 | 10 | 13 | 14 | 13 | 14 | 7 | 11 | 11 | 14 | 13 | 14 | 10 | 11 | 11 | 13 | 12 | 13 | 11 | 11 | 17 | 17 |
| 179 | 15 | 15 | 12 | 16 | 11 | 15 | 13 | 16 | 14 | 14 | 13 | 13 | 13 | 14 | 13 | 17 | 11 | 11 | 8 | 12 | 8 | 9 | 14 | 16 | 12 | 13 | 7 | 7 | 14 | 15 | 13 | 13 | 13 | 13 | 12 | 12 | 12 | 12 | 12 | 13 | 14 | 17 |
| 180 | 14 | 14 | 12 | 17 | 15 | 17 | 14 | 15 | 14 | 15 | 14 | 15 | 13 | 14 | 13 | 16 | 10 | 11 | 10 | 12 | 8 | 10 | 14 | 14 | 12 | 12 | 7 | 12 | 11 | 14 | 14 | 14 | 10 | 13 | 12 | 13 | 12 | 14 | 10 | 11 | 14 | 23 |
| 181 | 15 | 16 | 16 | 17 | 17 | 17 | 14 | 14 | 15 | 15 | 14 | 16 | 13 | 13 | 15 | 16 | 11 | 11.3 | 8 | 8 | 8 | 10 | 13.2 | 14 | 10 | 12 | 7 | 12 | 11 | 13 | 13 | 14 | 13 | 14 | 13 | 13 | 11 | 12 | 10 | 13 | 14 | 18 |
| 182 | 14 | 14 | 12 | 17 | 17 | 17 | 13 | 13 | 13 | 14 | 12 | 14 | 13 | 14 | 13 | 14 | 10 | 11 | 10 | 10 | 8 | 10 | 13.2 | 15.2 | 11 | 12 | 7 | 11 | 11 | 11 | 12 | 16 | 11 | 11 | 12 | 13 | 11 | 11 | 12 | 14 | 14 | 18 |
| 183 | 14 | 14 | 17 | 17 | 11 | 15 | 14 | 15 | 15 | 15 | 15 | 15 | 13 | 13 | 14 | 15 | 11 | 14 | 10 | 10 | 9 | 9 | 14 | 15.2 | 11 | 12 | 7 | 12 | 11 | 13 | 14 | 15 | 13 | 14 | 10 | 13 | 12 | 12 | 10.3 | 11 | 14 | 18 |
| 184 | 14 | 16 | 16 | 17 | 15 | 18 | 14 | 14 | 14 | 15 | 15 | 15 | 14 | 14 | 14 | 15 | 10 | 11 | 10 | 11 | 9 | 10 | 13 | 16.2 | 12 | 13 | 7 | 7 | 13 | 14 | 14 | 16 | 13 | 13 | 12 | 13 | 12 | 12 | 12 | 12 | 14 | 18 |
| 185 | 15 | 15 | 17 | 18 | 15 | 16 | 13 | 13 | 14 | 17 | 13 | 14 | 14 | 14 | 14 | 15 | 11 | 14 | 8 | 8 | 9 | 10 | 14 | 15 | 12 | 13 | 7 | 11 | 11 | 13 | 14 | 14 | 10 | 14 | 12 | 13 | 11 | 12 | 11 | 12 | 14 | 14 |
| 186 | 15 | 15 | 12 | 17 | 15 | 17 | 13 | 15 | 13 | 15 | 14 | 14 | 13 | 14 | 15 | 16 | 11 | 12 | 8 | 10 | 8 | 10 | 13.2 | 14 | 12 | 13 | 7 | 11 | 11 | 15 | 14 | 15 | 13 | 13 | 12 | 13 | 11 | 14 | 12 | 13 | 20 | 20 |
| 187 | 15 | 17 | 12 | 12 | 15 | 17 | 12 | 13 | 14 | 14 | 13 | 16 | 13 | 13 | 13 | 15 | 11 | 12 | 8 | 11 | 10 | 10 | 13.2 | 15.2 | 11 | 11 | 7 | 11 | 11 | 12 | 14 | 15 | 10 | 11 | 11 | 13 | 9 | 12 | 12 | 12 | 14 | 17 |
| 188 | 15 | 17 | 17 | 17 | 11 | 11 | 14 | 15 | 14 | 15 | 13 | 15 | 13 | 13 | 14 | 14 | 10 | 11 | 8 | 8 | 10 | 10 | 13 | 14 | 9 | 12 | 12 | 12 | 11 | 11 | 13 | 15 | 14 | 14 | 12 | 14 | 9 | 11 | 12 | 12 | 17 | 20 |
| 189 | 16 | 17 | 16 | 16 | 11 | 15 | 13 | 15 | 14 | 15 | 12 | 15 | 13 | 14 | 13 | 14 | 10 | 11.3 | 10 | 12 | 10 | 11 | 13 | 14.2 | 12 | 13 | 12 | 12 | 11 | 11 | 14 | 15 | 11 | 13 | 11 | 13 | 12 | 13 | 11 | 12 | 17 | 18 |
| 190 | 15 | 15 | 12 | 12 | 11 | 16 | 13 | 13 | 14 | 14 | 15 | 15 | 13 | 13 | 14 | 15 | 14 | 14 | 10 | 12 | 11 | 11 | 13 | 15.2 | 12 | 13 | 7 | 12 | 11 | 14 | 14 | 14 | 10 | 14 | 11 | 12 | 9 | 12 | 12 | 12 | 14 | 17 |
| 191 | 14 | 18 | 12 | 18 | 11 | 15 | 14 | 16 | 15 | 16 | 13 | 15 | 10 | 13 | 14 | 16 | 10 | 12 | 8 | 8 | 8 | 9 | 13 | 13 | 11 | 13 | 7 | 13 | 13 | 14 | 14 | 14 | 10 | 14 | 11 | 12 | 11 | 12 | 11 | 12 | 17 | 18 |
| 192 | 15 | 16 | 16 | 17 | 11 | 15 | 13 | 14 | 14 | 14 | 16 | 16 | 13 | 14 | 13 | 16 | 11 | 11.3 | 8 | 12 | 9 | 9 | 14 | 16 | 12 | 13 | 7 | 12 | 11 | 11 | 14 | 14 | 11 | 13 | 11 | 12 | 11 | 12 | 11 | 12 | 14 | 14 |
| 193 | 14 | 17 | 12 | 17 | 16 | 16 | 14 | 15 | 13 | 15 | 14 | 14 | 14 | 14 | 13 | 14 | 10 | 10 | 10 | 10 | 10 | 11 | 13 | 14.2 | 12 | 13 | 7 | 12 | 11 | 14 | 13 | 14 | 11 | 11 | 12 | 14 | 9 | 14 | 8 | 10 | 14 | 14 |
| 194 | 14 | 15 | 17 | 17 | 16 | 17 | 14 | 14 | 15 | 15 | 14 | 14 | 14 | 14 | 15 | 15 | 12 | 13 | 10 | 11 | 9 | 11 | 12.2 | 13 | 13 | 13 | 12 | 13 | 11 | 15 | 14 | 14 | 14 | 14 | 12 | 12 | 10 | 13 | 12 | 13 | 17 | 18 |
| 195 | 15 | 15 | 12 | 16 | 15 | 17 | 12 | 15 | 14 | 15 | 15 | 15 | 13 | 13 | 13 | 15 | 10 | 10 | 11 | 12 | 8 | 9 | 14 | 14 | 11 | 13 | 12 | 12 | 11 | 14 | 13 | 13 | 10 | 13 | 11 | 11 | 9 | 12 | 11 | 11 | 14 | 18 |
| 196 | 14 | 14 | 14 | 17 | 11 | 15 | 13 | 14 | 14 | 15 | 15 | 15 | 13 | 14 | 13 | 14 | 12 | 14 | 8 | 12 | 9 | 9 | 15.2 | 15.2 | 12 | 12 | 7 | 12 | 14 | 15 | 14 | 15 | 11 | 14 | 13 | 13 | 12 | 12 | 11 | 12 | 17 | 18 |
| 197 | 14 | 15 | 17 | 17 | 15 | 16 | 14 | 15 | 14 | 15 | 14 | 15 | 13 | 13 | 15 | 16 | 10 | 10 | 8 | 12 | 9 | 9 | 13 | 15.2 | 12 | 13 | 11 | 12 | 11 | 13 | 14 | 14 | 13 | 13 | 10 | 12 | 9 | 11 | 10 | 12 | 14 | 17 |
| 198 | 18 | 18 | 17 | 19 | 11 | 17 | 16 | 16 | 14 | 14 | 14 | 14 | 12 | 13 | 15 | 15 | 11 | 11 | 10 | 10 | 8 | 9 | 15.2 | 15.2 | 10 | 13 | 7 | 7 | 11 | 14 | 13 | 14 | 13 | 13 | 12 | 13 | 9 | 12 | 12 | 12 | 14 | 14 |
| 199 | 14 | 16 | 16 | 17 | 11 | 15 | 14 | 14 | 15 | 15 | 12 | 14 | 12 | 14 | 13 | 13 | 12 | 12 | 8 | 12 | 9 | 11 | 13 | 14.2 | 11 | 12 | 7 | 7 | 12 | 13 | 14 | 15 | 11 | 14 | 12 | 13 | 9 | 11 | 11 | 13 | 18 | 18 |
| 200 | 14 | 15 | 12 | 17 | 17 | 17 | 14 | 15 | 14 | 15 | 13 | 16 | 13 | 13 | 15 | 15 | 11 | 12 | 10 | 12 | 8 | 10 | 14 | 15 | 12 | 13 | 7 | 12 | 11 | 14 | 13 | 14 | 13 | 14 | 10 | 12 | 12 | 14 | 11 | 11 | 14 | 14 |
| 201 | 15 | 15 | 16 | 17 | 16 | 17 | 13 | 16 | 13 | 13 | 14 | 14 | 14 | 14 | 15 | 15 | 10 | 12 | 10 | 10 | 10 | 10 | 13 | 15.2 | 12 | 13 | 7 | 7 | 12 | 13 | 14 | 16 | 13 | 14 | 13 | 13 | 10 | 13 | 11 | 12 | 14 | 18 |
| 202 | 15 | 16 | 16 | 17 | 11 | 15 | 11 | 14 | 14 | 16 | 14 | 17 | 13 | 13 | 13 | 14 | 11 | 11 | 8 | 12 | 9 | 11 | 12 | 15.2 | 10 | 12 | 7 | 7 | 11 | 12 | 15 | 16 | 13 | 13 | 12 | 13 | 12 | 12 | 12 | 13 | 17 | 17 |
| 203 | 14 | 14 | 12 | 18 | 13 | 17 | 12 | 13 | 13 | 16 | 13 | 16 | 10 | 13 | 14 | 15 | 11 | 11 | 12 | 13 | 9 | 11 | 14 | 15.2 | 12 | 12 | 11 | 12 | 13 | 13 | 13 | 13 | 13 | 14 | 9 | 12 | 11 | 11 | 12 | 12 | 14 | 14 |
| 204 | 14 | 15 | 17 | 18 | 11 | 17 | 13 | 14 | 14 | 16 | 14 | 16 | 13 | 14 | 15 | 15 | 11 | 14 | 12 | 12 | 8 | 9 | 13 | 14 | 12 | 13 | 11 | 12 | 11 | 13 | 11 | 13 | 14 | 14 | 10 | 12 | 11 | 11 | 13 | 13 | 14 | 14 |
| 205 | 14 | 16 | 12 | 17 | 11 | 15 | 13 | 14 | 14 | 15 | 14 | 14 | 14 | 14 | 14 | 16 | 12 | 14 | 12 | 12 | 8 | 9 | 13 | 14.2 | 9 | 13 | 7 | 7 | 11 | 12 | 12 | 15 | 13 | 14 | 12 | 14 | 12 | 12 | 13 | 14 | 17 | 17 |
| Sample Name  Supplementary Table 1 (*Continued*)   |  |  |  |  |  |  |  |  |  |  |  |  |  |  |  |  |  |  |  |  |  |  |  |  |  |  |  |  |  |  |  |  |  |  |  |  |  |  |  |  |  |  |  | | --- | --- | --- | --- | --- | --- | --- | --- | --- | --- | --- | --- | --- | --- | --- | --- | --- | --- | --- | --- | --- | --- | --- | --- | --- | --- | --- | --- | --- | --- | --- | --- | --- | --- | --- | --- | --- | --- | --- | --- | --- | --- | --- | | D6S474 | D6S474 | D12ATA63 | D12ATA63 | D22S1045 | D22S1045 | D10S1248 | D10S1248 | D1S1677 | D1S1677 | D11S4463 | D11S4463 | D1S1627 | D1S1627 | D3S4529 | D3S4529 | D2S441 | D2S441 | D6S1017 | D6S1017 | D4S2408 | D4S2408 | D19S433 | D19S433 | D17S1301 | D17S1301 | D1GATA113 | D1GATA113 | D18S853 | D18S853 | D20S482 | D20S482 | D14S1434 | D14S1434 | D9S1122 | D9S1122 | D2S1776 | D2S1776 | D10S1435 | D10S1435 | D5S2500 | D5S2500 |
| 206 | 14 | 15 | 17 | 19 | 11 | 16 | 13 | 14 | 13 | 14 | 15 | 16 | 14 | 14 | 15 | 17 | 11.3 | 14 | 10 | 11 | 11 | 11 | 12 | 16.2 | 12 | 12 | 7 | 7 | 11 | 15 | 14 | 15 | 14 | 14 | 12 | 13 | 12 | 13 | 12 | 13 | 14 | 17 |
| 207 | 16 | 18 | 17 | 17 | 15 | 15 | 13 | 13 | 14 | 15 | 14 | 15 | 13 | 13 | 13 | 15 | 11 | 12 | 12 | 12 | 8 | 10 | 14.2 | 15.2 | 11 | 11 | 7 | 11 | 11 | 13 | 10 | 14 | 11 | 14 | 12 | 14 | 12 | 12 | 12 | 14 | 17 | 20 |
| 208 | 15 | 16 | 16 | 17 | 15 | 17 | 14 | 15 | 13 | 14 | 13 | 14 | 12 | 13 | 14 | 17 | 10 | 10 | 10 | 12 | 9 | 10 | 14 | 15.2 | 12 | 14 | 11 | 12 | 11 | 14 | 14 | 14 | 14 | 14 | 12 | 13 | 11 | 12 | 13 | 14 | 14 | 14 |
| 209 | 14 | 14 | 16 | 17 | 15 | 16 | 13 | 15 | 15 | 15 | 13 | 17 | 13 | 14 | 13 | 16 | 10 | 12 | 8 | 10 | 8 | 10 | 12 | 14 | 12 | 12 | 7 | 12 | 11 | 13 | 13 | 14 | 13 | 14 | 11 | 13 | 10 | 14 | 13 | 13 | 17 | 20 |
| 210 | 15 | 15 | 12 | 17 | 15 | 17 | 12 | 12 | 14 | 15 | 13 | 16 | 10 | 12 | 16 | 16 | 11 | 14 | 10 | 10 | 8 | 9 | 14 | 14.2 | 10 | 14 | 7 | 11 | 12 | 13 | 13 | 15 | 14 | 14 | 12 | 13 | 9 | 11 | 11 | 14 | 17 | 17 |
| 211 | 14 | 15 | 16 | 18 | 11 | 15 | 13 | 15 | 14 | 15 | 13 | 15 | 12 | 14 | 13 | 16 | 11 | 11 | 10 | 12 | 9 | 10 | 13 | 14 | 12 | 12 | 7 | 7 | 11 | 13 | 14 | 15 | 13 | 14 | 12 | 13 | 9 | 14 | 11 | 13 | 17 | 18 |
| 212 | 14 | 15 | 12 | 17 | 14 | 17 | 15 | 16 | 15 | 15 | 14 | 14 | 10 | 13 | 14 | 15 | 11 | 12 | 8 | 10 | 10 | 10 | 13 | 14.2 | 11 | 11 | 12 | 12 | 11 | 11 | 14 | 14 | 14 | 14 | 12 | 14 | 9 | 13 | 8 | 12 | 14 | 14 |
| 213 | 14 | 14 | 12 | 17 | 16 | 17 | 14 | 14 | 15 | 15 | 14 | 14 | 13 | 14 | 13 | 14 | 12 | 14 | 10 | 10 | 9 | 9 | 12 | 15 | 12 | 12 | 7 | 12 | 13 | 14 | 13 | 15 | 11 | 14 | 10 | 13 | 11 | 12 | 10 | 12 | 14 | 17 |
| 214 | 14 | 14 | 12 | 17 | 11 | 11 | 13 | 15 | 14 | 15 | 15 | 15 | 12 | 13 | 15 | 16 | 11 | 12 | 8 | 12 | 10 | 12 | 13 | 13.2 | 12 | 13 | 7 | 11 | 11 | 14 | 10 | 14 | 13 | 14 | 10 | 12 | 9 | 13 | 12 | 12 | 17 | 17 |
| 215 | 15 | 16 | 12 | 17 | 11 | 11 | 14 | 15 | 14 | 15 | 15 | 15 | 13 | 14 | 14 | 14 | 11 | 12 | 8 | 10 | 9 | 10 | 14 | 14 | 10 | 12 | 12 | 12 | 13 | 14 | 14 | 14 | 14 | 14 | 11 | 14 | 11 | 11 | 12 | 13 | 17 | 17 |
| 216 | 14 | 15 | 12 | 12 | 11 | 16 | 13 | 13 | 14 | 14 | 13 | 15 | 13 | 14 | 13 | 16 | 13 | 14 | 10 | 13 | 8 | 9 | 13.2 | 15 | 13 | 14 | 7 | 11 | 13 | 13 | 13 | 14 | 11 | 14 | 13 | 13 | 11 | 12 | 12 | 14 | 14 | 14 |
| 217 | 14 | 14 | 12 | 18 | 11 | 11 | 14 | 16 | 15 | 15 | 13 | 13 | 11 | 13 | 14 | 15 | 10 | 15 | 11 | 12 | 8 | 8 | 13 | 15.2 | 11 | 13 | 7 | 12 | 10 | 14 | 14 | 15 | 11 | 11 | 10 | 13 | 11 | 12 | 11 | 14 | 17 | 18 |
| 218 | 15 | 17 | 12 | 16 | 11 | 15 | 13 | 14 | 14 | 15 | 15 | 16 | 13 | 13 | 14 | 16 | 9.1 | 11 | 12 | 12 | 8 | 10 | 12 | 14 | 13 | 13 | 7 | 7 | 11 | 13 | 15 | 15 | 13 | 13 | 12 | 12 | 11 | 11 | 12 | 13 | 14 | 18 |
| 219 | 14 | 14 | 12 | 17 | 15 | 16 | 13 | 16 | 15 | 16 | 12 | 13 | 13 | 14 | 15 | 15 | 10 | 11 | 8 | 10 | 11 | 11 | 13 | 15 | 12 | 13 | 7 | 12 | 11 | 11 | 12 | 13 | 13 | 13 | 12 | 12 | 12 | 12 | 13 | 14 | 18 | 18 |
| 220 | 15 | 17 | 17 | 17 | 11 | 17 | 13 | 13 | 14 | 15 | 15 | 15 | 13 | 13 | 16 | 17 | 10 | 11 | 8 | 12 | 10 | 10 | 13 | 13 | 12 | 13 | 7 | 10 | 11 | 13 | 13 | 15 | 11 | 13 | 10 | 13 | 9 | 11 | 13 | 13 | 14 | 18 |
| 221 | 15 | 17 | 12 | 17 | 13 | 17 | 12 | 14 | 14 | 15 | 14 | 15 | 13 | 13 | 14 | 15 | 11 | 12 | 8 | 11 | 9 | 11 | 13 | 14 | 11 | 13 | 7 | 11 | 13 | 14 | 15 | 15 | 11 | 11 | 11 | 12 | 11 | 12 | 13 | 15 | 14 | 14 |
| 222 | 14 | 15 | 16 | 16 | 15 | 17 | 13 | 14 | 14 | 15 | 14 | 17 | 12 | 15 | 14 | 15 | 11 | 12 | 8 | 10 | 8 | 11 | 13 | 15.2 | 12 | 12 | 7 | 7 | 11 | 13 | 14 | 15 | 13 | 13 | 10 | 12 | 11 | 12 | 14 | 14 | 14 | 18 |
| 223 | 15 | 17 | 12 | 16 | 11 | 15 | 13 | 13 | 14 | 14 | 14 | 14 | 13 | 14 | 15 | 17 | 11 | 11 | 8 | 12 | 8 | 11 | 13.2 | 14 | 12 | 13 | 11 | 11 | 11 | 14 | 13 | 14 | 10 | 14 | 11 | 13 | 9 | 13 | 12 | 13 | 14 | 20 |
| 224 | 14 | 16 | 17 | 18 | 11 | 17 | 14 | 15 | 14 | 16 | 14 | 15 | 14 | 14 | 15 | 15 | 10 | 11 | 10 | 12 | 11 | 12 | 14 | 14.2 | 10 | 13 | 7 | 7 | 11 | 11 | 14 | 15 | 13 | 14 | 12 | 14 | 11 | 11 | 11 | 14 | 14 | 18 |
| 225 | 15 | 15 | 12 | 18 | 15 | 16 | 13 | 14 | 13 | 14 | 14 | 18 | 12 | 13 | 15 | 16 | 11 | 12 | 10 | 10 | 11 | 11 | 13.2 | 14.2 | 12 | 13 | 12 | 12 | 11 | 13 | 15 | 16 | 13 | 14 | 12 | 13 | 9 | 11 | 12 | 12 | 14 | 17 |
| 226 | 14 | 16 | 12 | 16 | 11 | 15 | 11 | 14 | 14 | 15 | 15 | 17 | 10 | 14 | 13 | 16 | 11 | 12 | 12 | 12 | 10 | 10 | 14 | 14 | 12 | 12 | 7 | 12 | 11 | 13 | 13 | 14 | 12 | 13 | 11 | 14 | 12 | 12 | 10 | 14 | 14 | 18 |
| 227 | 15 | 15 | 17 | 17 | 16 | 16 | 13 | 15 | 14 | 14 | 14 | 15 | 10 | 13 | 13 | 15 | 10 | 11 | 8 | 10 | 9 | 10 | 13 | 15.2 | 10 | 12 | 7 | 12 | 11 | 13 | 12 | 16 | 11 | 15 | 13 | 13 | 10 | 12 | 12 | 12 | 14 | 18 |
| 228 | 15 | 15 | 16 | 18 | 15 | 15 | 13 | 15 | 14 | 14 | 13 | 15 | 13 | 15 | 14 | 15 | 14 | 14 | 7 | 8 | 9 | 10 | 14.2 | 17 | 11 | 12 | 12 | 12 | 13 | 14 | 14 | 14 | 10 | 11 | 12 | 13 | 11 | 13 | 11 | 13 | 14 | 18 |
| 229 | 14 | 14 | 12 | 12 | 17 | 17 | 13 | 15 | 14 | 14 | 15 | 15 | 13 | 13 | 15 | 17 | 10 | 12 | 10 | 10 | 9 | 10 | 13 | 13.2 | 12 | 14 | 7 | 11 | 14 | 15 | 10 | 13 | 14 | 14 | 12 | 15 | 12 | 13 | 11 | 14 | 14 | 20 |
| 230 | 15 | 18 | 16 | 16 | 11 | 16 | 14 | 14 | 15 | 15 | 14 | 15 | 13 | 13 | 14 | 15 | 10 | 11 | 8 | 13 | 8 | 9 | 13.2 | 14 | 10 | 13 | 7 | 12 | 11 | 14 | 13 | 16 | 14 | 14 | 14 | 16 | 10 | 12 | 12 | 13 | 14 | 18 |
| 231 | 16 | 17 | 12 | 17 | 11 | 16 | 13 | 15 | 14 | 14 | 12 | 13 | 12 | 13 | 15 | 16 | 10 | 11 | 13 | 13 | 9 | 10 | 13 | 14 | 12 | 12 | 7 | 12 | 11 | 13 | 10 | 14 | 10 | 13 | 11 | 12 | 9 | 12 | 8 | 13 | 17 | 23 |
| 232 | 14 | 15 | 12 | 14 | 11 | 16 | 13 | 15 | 15 | 15 | 13 | 14 | 13 | 13 | 14 | 15 | 10 | 11 | 12 | 12 | 8 | 9 | 14 | 14 | 12 | 12 | 7 | 12 | 13 | 14 | 14 | 15 | 11 | 13 | 13 | 13 | 9 | 12 | 10 | 11 | 14 | 14 |
| 233 | 15 | 15 | 12 | 12 | 11 | 11 | 13 | 15 | 13 | 14 | 13 | 16 | 12 | 12 | 14 | 15 | 10 | 14 | 10 | 12 | 9 | 10 | 13 | 13.2 | 12 | 12 | 11 | 11 | 11 | 14 | 12 | 15 | 11 | 13 | 10 | 13 | 11 | 12 | 10 | 12 | 17 | 18 |
| 234 | 14 | 14 | 16 | 17 | 11 | 17 | 13 | 15 | 14 | 15 | 14 | 15 | 10 | 13 | 13 | 15 | 10 | 11 | 10 | 12 | 8 | 11 | 13 | 14.2 | 11 | 12 | 7 | 12 | 11 | 11 | 13 | 15 | 11 | 13 | 12 | 13 | 11 | 11 | 12 | 14 | 14 | 18 |
| 235 | 16 | 17 | 12 | 17 | 15 | 15 | 12 | 15 | 14 | 14 | 13 | 14 | 12 | 12 | 15 | 15 | 11 | 11 | 8 | 13 | 10 | 10 | 13 | 14.2 | 10 | 13 | 7 | 12 | 14 | 14 | 12 | 13 | 11 | 14 | 10 | 11 | 12 | 12 | 11 | 12 | 14 | 18 |
| 236 | 14 | 14 | 16 | 17 | 11 | 15 | 13 | 13 | 10 | 13 | 14 | 15 | 13 | 14 | 13 | 14 | 12 | 12 | 10 | 12 | 9 | 11 | 14 | 14.2 | 12 | 12 | 12 | 12 | 11 | 11 | 14 | 15 | 12 | 13 | 10 | 11 | 10 | 12 | 11 | 12 | 17 | 18 |
| 237 | 14 | 16 | 12 | 17 | 16 | 17 | 13 | 14 | 13 | 15 | 13 | 14 | 13 | 13 | 13 | 15 | 12 | 12 | 12 | 12 | 8 | 9 | 13.2 | 14 | 10 | 12 | 11 | 11 | 11 | 14 | 14 | 15 | 11 | 15 | 13 | 13 | 9 | 12 | 11 | 12 | 17 | 18 |
| 238 | 14 | 15 | 12 | 17 | 11 | 15 | 15 | 15 | 14 | 14 | 14 | 15 | 13 | 13 | 13 | 15 | 12 | 12 | 10 | 13 | 10 | 10 | 13 | 15.2 | 12 | 12 | 7 | 13 | 12 | 15 | 10 | 14 | 11 | 14 | 12 | 13 | 11 | 12 | 12 | 13 | 14 | 18 |
| 239 | 14 | 15 | 17 | 20 | 15 | 16 | 13 | 14 | 14 | 15 | 14 | 14 | 13 | 14 | 13 | 16 | 11 | 14 | 12 | 12 | 8 | 11 | 13 | 14 | 9 | 12 | 7 | 7 | 11 | 11 | 13 | 14 | 13 | 14 | 12 | 13 | 9 | 9 | 11 | 11 | 17 | 18 |
| 240 | 17 | 17 | 17 | 17 | 11 | 16 | 13 | 14 | 14 | 16 | 14 | 14 | 10 | 14 | 14 | 15 | 10 | 11 | 10 | 12 | 9 | 10 | 12.2 | 14 | 11 | 12 | 7 | 12 | 11 | 13 | 13 | 16 | 11 | 15 | 12 | 13 | 11 | 13 | 12 | 13 | 18 | 23 |
| 241 | 14 | 16 | 17 | 17 | 16 | 18 | 14 | 15 | 13 | 14 | 14 | 14 | 13 | 14 | 13 | 15 | 10 | 12 | 8 | 12 | 10 | 11 | 13.2 | 14.2 | 11 | 13 | 7 | 7 | 13 | 14 | 13 | 16 | 14 | 15 | 11 | 13 | 9 | 12 | 12 | 12 | 14 | 20 |
| 242 | 15 | 17 | 12 | 12 | 11 | 11 | 13 | 14 | 14 | 14 | 14 | 16 | 13 | 14 | 13 | 14 | 11 | 12 | 10 | 13 | 8 | 9 | 13 | 13 | 12 | 12 | 7 | 7 | 11 | 13 | 13 | 14 | 14 | 14 | 10 | 12 | 12 | 13 | 11 | 11 | 14 | 14 |
| 243 | 16 | 16 | 17 | 17 | 15 | 16 | 13 | 14 | 14 | 14 | 14 | 14 | 13 | 13 | 14 | 14 | 10 | 12 | 8 | 12 | 8 | 10 | 13 | 14 | 11 | 13 | 7 | 7 | 13 | 14 | 13 | 14 | 13 | 13 | 13 | 13 | 9 | 12 | 10 | 11 | 17 | 18 |
| 244 | 14 | 15 | 12 | 14 | 11 | 17 | 13 | 14 | 13 | 14 | 12 | 13 | 12 | 13 | 14 | 15 | 12 | 12 | 11 | 12 | 9 | 10 | 13 | 13 | 12 | 13 | 7 | 12 | 11 | 13 | 12 | 14 | 10 | 14 | 9 | 13 | 12 | 14 | 12 | 12 | 14 | 18 |
| 245 | 14 | 15 | 18 | 18 | 11 | 17 | 13 | 13 | 15 | 15 | 14 | 15 | 13 | 13 | 13 | 15 | 11 | 12 | 8 | 10 | 10 | 11 | 14 | 15 | 11 | 13 | 7 | 7 | 11 | 14 | 13 | 16 | 11 | 14 | 11 | 12 | 10 | 11 | 11 | 14 | 14 | 17 |
| 246 | 15 | 16 | 12 | 14 | 15 | 16 | 14 | 15 | 14 | 14 | 14 | 17 | 13 | 13 | 15 | 15 | 10 | 14 | 10 | 10 | 9 | 10 | 13 | 13 | 12 | 12 | 7 | 7 | 11 | 14 | 14 | 15 | 14 | 14 | 11 | 12 | 11 | 12 | 12 | 13 | 14 | 17 |
| Sample Name  Supplementary Table 1 (*Continued*)   |  |  |  |  |  |  |  |  |  |  |  |  |  |  |  |  |  |  |  |  |  |  |  |  |  |  |  |  |  |  |  |  |  |  |  |  |  |  |  |  |  |  |  | | --- | --- | --- | --- | --- | --- | --- | --- | --- | --- | --- | --- | --- | --- | --- | --- | --- | --- | --- | --- | --- | --- | --- | --- | --- | --- | --- | --- | --- | --- | --- | --- | --- | --- | --- | --- | --- | --- | --- | --- | --- | --- | --- | | D6S474 | D6S474 | D12ATA63 | D12ATA63 | D22S1045 | D22S1045 | D10S1248 | D10S1248 | D1S1677 | D1S1677 | D11S4463 | D11S4463 | D1S1627 | D1S1627 | D3S4529 | D3S4529 | D2S441 | D2S441 | D6S1017 | D6S1017 | D4S2408 | D4S2408 | D19S433 | D19S433 | D17S1301 | D17S1301 | D1GATA113 | D1GATA113 | D18S853 | D18S853 | D20S482 | D20S482 | D14S1434 | D14S1434 | D9S1122 | D9S1122 | D2S1776 | D2S1776 | D10S1435 | D10S1435 | D5S2500 | D5S2500 |
| 247 | 14 | 15 | 16 | 18 | 11 | 15 | 14 | 15 | 14 | 15 | 13 | 14 | 13 | 13 | 14 | 16 | 10 | 11 | 10 | 13 | 10 | 10 | 13 | 13.2 | 10 | 12 | 12 | 12 | 11 | 14 | 13 | 14 | 10 | 14 | 12 | 13 | 11 | 12 | 11 | 13 | 14 | 17 |
| 248 | 14 | 16 | 12 | 12 | 15 | 16 | 15 | 16 | 14 | 15 | 12 | 14 | 10 | 12 | 15 | 17 | 10 | 15 | 8 | 10 | 9 | 11 | 12 | 14 | 10 | 12 | 11 | 12 | 11 | 13 | 14 | 14 | 11 | 13 | 12 | 12 | 10 | 11 | 12 | 13 | 17 | 17 |
| 249 | 14 | 17 | 16 | 17 | 15 | 16 | 13 | 13 | 14 | 15 | 13 | 13 | 13 | 14 | 15 | 17 | 10 | 10 | 11 | 12 | 8 | 9 | 14 | 15.2 | 12 | 12 | 7 | 12 | 11 | 13 | 14 | 14 | 13 | 13 | 12 | 13 | 11 | 13 | 13 | 13 | 17 | 17 |
| 250 | 14 | 14 | 17 | 17 | 15 | 16 | 13 | 13 | 13 | 14 | 13 | 14 | 12 | 13 | 13 | 14 | 10 | 12 | 8 | 10 | 9 | 9 | 12 | 13 | 10 | 12 | 11 | 12 | 13 | 14 | 10 | 13 | 13 | 14 | 10 | 10 | 9 | 12 | 11 | 12 | 17 | 17 |
| 251 | 14 | 15 | 12 | 14 | 11 | 15 | 15 | 16 | 14 | 14 | 15 | 15 | 13 | 14 | 14 | 16 | 11.3 | 14 | 10 | 12 | 8 | 8 | 12 | 14 | 12 | 13 | 7 | 7 | 11 | 13 | 12 | 14 | 13 | 13 | 13 | 14 | 11 | 12 | 13 | 14 | 14 | 17 |
| 252 | 14 | 15 | 12 | 16 | 16 | 17 | 16 | 17 | 14 | 15 | 12 | 14 | 13 | 14 | 12 | 14 | 11 | 11 | 10 | 12 | 8 | 9 | 13 | 13.2 | 12 | 12 | 7 | 7 | 11 | 14 | 14 | 15 | 14 | 14 | 13 | 13 | 9 | 12 | 11 | 13 | 14 | 14 |
| 253 | 14 | 15 | 17 | 17 | 17 | 17 | 13 | 13 | 14 | 15 | 13 | 14 | 12 | 13 | 15 | 15 | 9.1 | 15 | 11 | 13 | 10 | 11 | 13 | 14 | 11 | 12 | 7 | 7 | 11 | 14 | 13 | 15 | 13 | 14 | 10 | 12 | 11 | 12 | 13 | 14 | 14 | 14 |
| 254 | 14 | 15 | 12 | 18 | 16 | 17 | 12 | 12 | 14 | 14 | 9 | 15 | 13 | 13 | 14 | 15 | 12 | 14 | 8 | 10 | 8 | 12 | 13 | 13.2 | 13 | 13 | 7 | 13 | 11 | 13 | 14 | 14 | 10 | 11 | 13 | 16 | 9 | 10 | 12 | 14 | 14 | 17 |
| 255 | 14 | 14 | 12 | 17 | 16 | 16 | 12 | 13 | 13 | 15 | 13 | 15 | 13 | 13 | 13 | 14 | 11 | 11.3 | 10 | 11 | 8 | 10 | 14 | 14 | 8 | 12 | 11 | 12 | 11 | 11 | 13 | 15 | 13 | 14 | 13 | 13 | 9 | 12 | 14 | 14 | 14 | 18 |
| 256 | 14 | 17 | 17 | 18 | 15 | 16 | 13 | 13 | 13 | 14 | 13 | 16 | 13 | 13 | 13 | 16 | 10 | 11 | 8 | 12 | 9 | 9 | 14 | 15.2 | 10 | 11 | 7 | 13 | 11 | 13 | 12 | 13 | 14 | 14 | 12 | 12 | 11 | 12 | 11 | 14 | 14 | 18 |
| 257 | 15 | 17 | 12 | 16 | 15 | 16 | 11 | 16 | 14 | 14 | 15 | 17 | 12 | 13 | 15 | 15 | 10 | 12 | 10 | 12 | 10 | 10 | 13 | 14 | 11 | 14 | 11 | 12 | 12 | 14 | 12 | 14 | 10 | 14 | 10 | 14 | 12 | 13 | 12 | 13 | 17 | 17 |
| 258 | 17 | 18 | 16 | 16 | 11 | 16 | 12 | 14 | 16 | 16 | 13 | 15 | 10 | 12 | 15 | 15 | 10 | 12 | 10 | 13 | 11 | 11 | 14 | 14 | 13 | 13 | 7 | 11 | 11 | 14 | 13 | 14 | 13 | 13 | 12 | 13 | 10 | 12 | 12 | 13 | 14 | 14 |
| 259 | 15 | 17 | 18 | 18 | 11 | 15 | 13 | 14 | 14 | 14 | 15 | 15 | 14 | 14 | 14 | 14 | 11 | 11 | 8 | 10 | 10 | 11 | 14 | 14 | 10 | 11 | 7 | 11 | 11 | 13 | 14 | 14 | 10 | 13 | 13 | 13 | 11 | 11 | 8 | 13 | 18 | 18 |
| 260 | 14 | 15 | 16 | 17 | 11 | 15 | 13 | 13 | 14 | 15 | 13 | 15 | 13 | 13 | 16 | 16 | 13 | 14 | 10 | 14 | 9 | 11 | 14 | 14 | 12 | 13 | 7 | 11 | 13 | 14 | 14 | 15 | 10 | 11 | 13 | 13 | 10 | 12 | 12 | 13 | 14 | 20 |
| 261 | 14 | 14 | 12 | 17 | 15 | 17 | 13 | 13 | 14 | 14 | 13 | 14 | 13 | 14 | 14 | 15 | 11 | 12 | 8 | 10 | 9 | 9 | 13 | 15 | 12 | 12 | 7 | 13 | 11 | 14 | 11 | 16 | 14 | 15 | 12 | 14 | 11 | 12 | 12 | 13 | 14 | 20 |
| 262 | 15 | 15 | 14 | 19 | 16 | 17 | 13 | 14 | 14 | 15 | 15 | 16 | 14 | 14 | 15 | 15 | 10 | 11 | 8 | 14 | 8 | 9 | 15 | 15.2 | 12 | 12 | 11 | 11 | 14 | 14 | 14 | 15 | 14 | 14 | 13 | 14 | 12 | 13 | 13 | 13 | 18 | 23 |
| 263 | 14 | 18 | 16 | 16 | 11 | 15 | 13 | 13 | 14 | 15 | 13 | 13 | 10 | 13 | 15 | 17 | 11 | 14 | 10 | 10 | 10 | 11 | 13 | 14.2 | 12 | 13 | 7 | 7 | 11 | 13 | 14 | 15 | 13 | 14 | 12 | 13 | 10 | 11 | 10 | 11 | 17 | 20 |
| 264 | 14 | 18 | 12 | 12 | 15 | 15 | 12 | 15 | 14 | 15 | 15 | 15 | 13 | 14 | 13 | 15 | 10 | 14.1 | 10 | 12 | 9 | 9 | 14 | 14.2 | 12 | 13 | 7 | 7 | 15 | 15 | 12 | 14 | 14 | 15 | 10 | 11 | 11 | 12 | 12 | 13 | 17 | 18 |
| 265 | 14 | 17 | 12 | 17 | 14 | 16 | 13 | 15 | 13 | 15 | 13 | 13 | 13 | 13 | 13 | 16 | 11 | 12 | 8 | 10 | 8 | 10 | 14 | 15.2 | 11 | 12 | 11 | 12 | 14 | 14 | 14 | 15 | 11 | 11 | 11 | 13 | 9 | 10 | 12 | 13 | 17 | 18 |
| 266 | 14 | 17 | 12 | 17 | 16 | 16 | 13 | 17 | 13 | 14 | 14 | 16 | 13 | 14 | 16 | 16 | 11 | 11 | 10 | 10 | 10 | 10 | 14.2 | 15 | 13 | 13 | 7 | 7 | 13 | 13 | 14 | 14 | 13 | 14 | 12 | 14 | 11 | 12 | 11 | 12 | 14 | 14 |
| 267 | 15 | 15 | 12 | 18 | 15 | 16 | 12 | 12 | 14 | 15 | 14 | 16 | 13 | 16 | 15 | 15 | 13 | 14 | 10 | 12 | 8 | 9 | 13 | 13 | 10 | 12 | 11 | 11 | 11 | 13 | 14 | 15 | 13 | 14 | 13 | 13 | 12 | 14 | 12 | 14 | 14 | 17 |
| 268 | 14 | 16 | 17 | 17 | 11 | 15 | 15 | 15 | 15 | 15 | 12 | 15 | 13 | 13 | 15 | 15 | 10 | 14 | 12 | 13 | 10 | 11 | 14.2 | 15.2 | 11 | 12 | 7 | 12 | 11 | 14 | 12 | 16 | 14 | 14 | 11 | 13 | 11 | 12 | 12 | 13 | 14 | 17 |
| 269 | 14 | 14 | 12 | 12 | 16 | 17 | 14 | 14 | 14 | 14 | 13 | 13 | 13 | 14 | 14 | 15 | 11 | 12 | 10 | 10 | 9 | 10 | 14 | 14.2 | 9 | 11 | 11 | 12 | 11 | 13 | 14 | 15 | 11 | 13 | 13 | 13 | 9 | 12 | 12 | 13 | 14 | 18 |
| 270 | 16 | 16 | 11 | 17 | 15 | 17 | 13 | 15 | 14 | 14 | 14 | 17 | 12 | 14 | 15 | 16 | 11 | 11 | 10 | 12 | 9 | 9 | 14 | 16.2 | 11 | 12 | 7 | 12 | 11 | 15 | 13 | 14 | 10 | 14 | 11 | 11 | 12 | 12 | 11 | 13 | 14 | 18 |
| 271 | 14 | 15 | 12 | 16 | 11 | 15 | 13 | 15 | 14 | 14 | 12 | 15 | 12 | 14 | 13 | 16 | 12 | 14 | 13 | 13 | 8 | 9 | 12 | 15.2 | 13 | 14 | 7 | 7 | 13 | 14 | 13 | 14 | 10 | 10 | 12 | 13 | 10 | 11 | 12 | 13 | 14 | 18 |
| 272 | 15 | 16 | 17 | 17 | 14 | 16 | 14 | 16 | 13 | 14 | 12 | 13 | 13 | 13 | 15 | 15 | 11 | 14 | 12 | 13 | 9 | 11 | 13 | 13 | 13 | 13 | 11 | 11 | 11 | 14 | 14 | 14 | 12 | 14 | 12 | 13 | 12 | 12 | 13 | 14 | 14 | 18 |
| 273 | 16 | 16 | 12 | 12 | 15 | 17 | 14 | 15 | 14 | 14 | 14 | 15 | 13 | 14 | 15 | 15 | 11 | 11.3 | 10 | 12 | 8 | 10 | 14.2 | 14.2 | 12 | 12 | 7 | 11 | 11 | 15 | 13 | 15 | 10 | 13 | 10 | 14 | 11 | 12 | 12 | 13 | 17 | 18 |
| 274 | 15 | 15 | 17 | 17 | 11 | 15 | 12 | 14 | 14 | 14 | 15 | 16 | 11 | 13 | 13 | 16 | 11 | 13 | 8 | 12 | 8 | 11 | 13 | 14 | 11 | 12 | 7 | 11 | 11 | 13 | 10 | 15 | 10 | 15 | 11 | 13 | 9 | 11 | 13 | 13 | 14 | 20 |
| 275 | 14 | 14 | 12 | 12 | 11 | 15 | 13 | 15 | 14 | 14 | 12 | 13 | 13 | 13 | 15 | 15 | 10 | 11 | 8 | 12 | 8 | 10 | 14.2 | 15.2 | 9 | 11 | 7 | 11 | 11 | 11 | 14 | 14 | 14 | 14 | 12 | 13 | 11 | 14 | 10 | 12 | 14 | 18 |

Supplementary Table 2. The p-values of linkage disequilibrium tests among 21 loci of Guanzhong Han population, China.

| locus | D10S1248 | D10S1435 | D11S4463 | D12ATA63 | D14S1434 | D17S1301 | D18S853 | D19S433 | D1GATA113 | D1S1677 | D20S482 | D22S1045 | D2S1776 | D2S441 | D3S4529 | D4S2408 | D5S2500 | D6S1017 | D6S474 | D9S1122 |
| --- | --- | --- | --- | --- | --- | --- | --- | --- | --- | --- | --- | --- | --- | --- | --- | --- | --- | --- | --- | --- |
| D10S1435 | 0.3300 |  |  |  |  |  |  |  |  |  |  |  |  |  |  |  |  |  |  |  |
| D11S4463 | 0.4031 | 0.3258 |  |  |  |  |  |  |  |  |  |  |  |  |  |  |  |  |  |  |
| D12ATA63 | 0.6405 | 0.7908 | 0.4167 |  |  |  |  |  |  |  |  |  |  |  |  |  |  |  |  |  |
| D14S1434 | 0.5075 | 0.4826 | 0.3373 | 0.7420 |  |  |  |  |  |  |  |  |  |  |  |  |  |  |  |  |
| D17S1301 | 0.8377 | 0.7323 | 0.9733 | 0.4222 | 0.4031 |  |  |  |  |  |  |  |  |  |  |  |  |  |  |  |
| D18S853 | 0.0686 | 0.8436 | 0.7690 | 0.4810 | 0.6036 | 0.2230 |  |  |  |  |  |  |  |  |  |  |  |  |  |  |
| D19S433 | 0.9015 | 0.3668 | 0.2062 | 0.3269 | 0.5076 | 0.3258 | 0.0146 |  |  |  |  |  |  |  |  |  |  |  |  |  |
| D1GATA113 | 0.5112 | 0.6765 | 0.7023 | 0.3252 | 0.6454 | 0.9755 | 0.1513 | 0.6973 |  |  |  |  |  |  |  |  |  |  |  |  |
| D1S1677 | 0.5784 | 0.8749 | 0.9780 | 0.7187 | 0.1601 | 0.1792 | 0.4612 | 0.2457 | 0.1596 |  |  |  |  |  |  |  |  |  |  |  |
| D20S482 | 0.6482 | 0.0589 | 0.6491 | 0.9108 | 0.3266 | 0.4576 | 0.9321 | 0.4805 | 0.0703 | 0.1555 |  |  |  |  |  |  |  |  |  |  |
| D22S1045 | 0.5066 | 0.3858 | 0.9590 | 0.1696 | 0.2989 | 0.0340 | 0.0200 | 0.1081 | 0.2575 | 0.9745 | 0.1577 |  |  |  |  |  |  |  |  |  |
| D2S1776 | 0.3104 | 0.0438 | 0.2746 | 0.7245 | 0.9751 | 0.9188 | 0.8849 | 0.2938 | 0.1076 | 0.5154 | 0.8702 | 0.8003 |  |  |  |  |  |  |  |  |
| D2S441 | 0.1579 | 0.8532 | 0.9876 | 0.3631 | 0.2000 | 0.1981 | 0.7795 | 0.8118 | 0.3018 | 0.9455 | 0.2793 | 0.0413 | 0.8097 |  |  |  |  |  |  |  |
| D3S4529 | 0.1893 | 0.5689 | 0.9343 | 0.8328 | 0.2410 | 0.4833 | 0.0115 | 0.5152 | 0.8265 | 0.9917 | 0.5679 | 0.4211 | 0.0269 | 0.1804 |  |  |  |  |  |  |
| D4S2408 | 0.3850 | 0.8644 | 0.6043 | 0.0744 | 0.4767 | 0.9860 | 0.9271 | 0.1409 | 0.1727 | 0.2757 | 0.5022 | 0.2608 | 0.1715 | 0.9770 | 0.9709 |  |  |  |  |  |
| D5S2500 | 0.8828 | 0.5574 | 0.5731 | 0.6477 | 0.8151 | 0.7741 | 0.2065 | 0.2050 | 0.6629 | 0.3764 | 0.9855 | 0.1147 | 0.6644 | 0.9101 | 0.4320 | 0.3470 |  |  |  |  |
| D6S1017 | 0.7163 | 0.3148 | 0.4108 | 0.7456 | 0.2189 | 0.4705 | 0.4188 | 0.5296 | 0.5790 | 0.4447 | 0.5440 | 0.2727 | 0.1755 | 0.8953 | 0.8152 | 0.8830 | 0.2084 |  |  |  |
| D6S474 | 0.7389 | 0.6394 | 0.2664 | 0.1744 | 0.8150 | 0.0128 | 0.4559 | 0.3379 | 0.8572 | 0.4218 | 0.5069 | 0.8538 | 0.9242 | 0.0425 | 0.9599 | 0.0410 | 0.7885 | 0.6127 |  |  |
| D9S1122 | 0.2995 | 0.1919 | 0.5133 | 0.0615 | 0.6603 | 0.4396 | 0.9716 | 0.6280 | 0.3419 | 0.6343 | 0.0570 | 0.3002 | 0.6450 | 0.6326 | 0.3752 | 0.2718 | 0.0612 | 0.3860 | 0.2654 |  |
| D1S1627 | 0.0307 | 0.2538 | 0.2892 | 0.5832 | 0.3077 | 0.7427 | 0.1389 | 0.8361 | 0.1399 | 0.1754 | 0.5940 | 0.2673 | 0.0571 | 0.6930 | 0.4120 | 0.6875 | 0.5613 | 0.3599 | 0.2903 | 0.3938 |
